# Genome-wide bioinformatics analysis of the *MATE* gene family for abiotic stress tolerance in sunflower (*Helianthus annuus* L.)

**DOI:** 10.1371/journal.pone.0346769

**Published:** 2026-04-13

**Authors:** Mohammad Nazmol Hasan, Md. Robin Islam, Rafee Shahrier, Md. Bayazid Hossen

**Affiliations:** 1 Department of Agricultural and Applied Statistics, Gazipur Agricultural University, Gazipur, Bangladesh; 2 Department of Genetics and Plant Breeding, Gazipur Agricultural University, Gazipur, Bangladesh; 3 Plant Breeding Division, Bangladesh Rice Research Institute, Gazipur, Bangladesh; 4 Department of Agricultural and Applied Statistics, Bangladesh Agricultural University, Mymensingh, Bangladesh; 5 Department of Agricultural Statistics, Sher-e-Bangla Agricultural University, Dhaka, Bangladesh; University of Delhi, INDIA

## Abstract

Abiotic stressors, such as drought, salinity, and heavy metals, induce physiological changes, nutritional imbalances, molecular alterations, and oxidative stress in plants, which significantly reduce productivity. However, the secondary transporters, multidrug and toxic compound extrusion (*MATE*) proteins, transport substrates and metabolites. Accordingly, in response to abiotic stressors, these proteins strengthen plants’ immune systems, detoxify toxins, and enhance growth and development. Although the roles of *MATE* proteins responding to abiotic stresses have been investigated in several plants, their functions in sunflower have not yet been discovered. Therefore, this study identified 74 *MATE* proteins in sunflower (*HanMATE*) based on phylogenetic analysis, which were distributed into four subgroups. Their *MATE*-like properties were then validated using the domain, motif, gene structure, gene duplication, and physicochemical analysis. The *HanMATE* proteins in various cell organelles play a crucial role in abiotic stress tolerance, scavenging reactive oxygen species (ROS), and regulating transcription. Subsequently, Most *HanMATE* genes are enriched with biological processes and molecular functions that transport micro- and macro-molecules, drugs, negatively charged ions, organic anions, and citrate. The important *Cis*-regulatory elements (CREs), abscisic acid-, light-, and MeJA-responsive elements in *HanMATE* genes regulate plants’ growth and development in stress conditions. The synteny analysis indicated that 41 *HanMATE* proteins exhibit over 75% sequence similarity with 40 established stress-responsive (SR) *MATE* proteins from various plant species, suggesting their potential SR characteristics. Furthermore, this study identified 136 microRNAs linked to 58 *HanMATE* proteins, including 19 major hub microRNAs and 31 hub *HanMATE* proteins, which may enhance sunflower agronomic traits and abiotic stress resistance. The *HanMATE* proteins are conserved in other species that contribute to detoxification and have stable binding affinity with flavonoids and citric acid, validated from 3D structural modeling, molecular docking (MD), dynamic simulation, and functional prediction. These findings demonstrate that *HanMATE* genes are essential for sunflower abiotic stress tolerance (AST), and genetic engineering can be applied to develop more robust sunflower.

## 1. Introduction

Climate change brings about various environmental changes, which result in abiotic stresses in plants, including drought, salinity, heavy metal stress, and extreme temperatures [1,2]. Plants’ stationary nature significantly intensifies their vulnerability, affecting growth, reproduction, and productivity [3–5]. Among the abiotic stresses, drought occurs more frequently and with greater severity. It exposes plants to closing their stomata, reducing transpiration, limiting photosynthesis, causing nutrient imbalance, and enhancing ROS production in different cellular compartments, ultimately bringing the plant under salt stress [6–9]. High salinity, resulting from excessive concentrations of Na^+^ and Cl^-^ in soil, causes high salinity and creates hyperosmotic and hyperionic conditions. These circumstances impede plant water and nutrient absorption, causing ionic and osmotic stresses, stomatal closure, limiting CO₂ uptake and photosynthesis, and oxidative stress [10–13]. Heavy metals, such as copper (Cu), cadmium (Cd), lead (Pb), iron (Fe), molybdenum (Mo), zinc (Zn), nickel (Ni), manganese (Mn), and cobalt (Co), are essential micronutrients for plant growth and development [14]. However, their excessive uptake can cause toxic effects, including inducing water-passive stomatal closure, impairing nutrient uptake channels, and excessive production of ROS in plants [15–22]. Furthermore, aluminum (AL) toxicity affects plants by inhibiting root growth and branching, binding to phosphorus, competing with essential cations, causing oxidative stress, and altering gene expression. It also affects signal transduction pathways, impacting plant responses to stress [23,24]. In conclusion, plants react to these stressors in comparable physiological and molecular ways, such as osmotic imbalance, cell dehydration, altered gene expression, and ROS generation [16,21–23,25,26].

The *MATE* proteins are secondary transporters primarily responsible for transporting many substrates, including chemical molecules, secondary metabolites, and phytohormones. Thus, *MATE* proteins detoxify internal and external toxins while simultaneously promoting the growth and development of plants. The *MATE* members are present in both prokaryotes and eukaryotes, and they are essential for multiple biological activities [27,28]. The *MATE* proteins comprise 400–700 amino acids and have a 40% sequence similarity among twelve transmembrane helices [27–29]. In mammals, *MATE* proteins were first identified in humans and mice. The *SLC47A1* and *SLC47A2* genes encode the human *MATE1* and *MATE2* proteins, which are mostly expressed in the kidney and liver [30–32]. Mammalian *MATE* proteins act as multispecific, electron-neutral organic cation transporters, facilitating the discharge of various organic cations and cationic therapeutics [33,34]. However, the *MATE* family is more diverse in plants than bacteria and animals [35]. In *A. thaliana*, the *MATE* gene *AtDTX1* plays a crucial role in transporting and detoxifying alkaloids, antibiotics, and heavy metals such as Cd [36]. A mutation in *AtDTX50* causes more abscisic acid (ABA) accumulation, upregulates many ABA marker genes, and is critical for ABA-mediated growth inhibition and drought stress responses [37]. In diploid cotton, *GrMATE18/34/41/51* were considerably upregulated in response to stress from drought, salt, and Cd; these genes may be suitable candidates for breeders looking to produce more AST genotypes [38]. Khan et al. [39] discovered that *HuMATE7/11/12/28* genes may aid in plant toxin detoxification from heavy metals and high soil salinity, paving the way for AST dragon fruit development. The *cis*-elements and expression pattern analysis identified *GmMATE75* as a candidate gene for AL tolerance in soybean [40]. *OsMATE4* and *OsMATE9* are likely involved in the physiological process of transporting and detoxifying metal ions [41]. In potato, *StMATE18/60/40/33/5* and *StMATE33* were significantly upregulated by Cu_2_^+^ and Cd_2_^+^ stress, respectively, while *StMATE59* was induced considerably by Ni_2_^+^ stress [42]. Therefore, *MATE* transporters play a crucial role in how plants respond to various abiotic stresses, including drought, salt, and heavy metal toxicity. Additionally, stress-resilient plants might be developed utilizing biotechnological approaches to *MATE* genes.

The sunflower is moderately abiotic stress-tolerant due to the special structure of its vital organs: the root, stem, leaves, and head [43,44]. Thereby, the abiotic stress tolerance of sunflower has been studied in many approaches, such as controlling specialized metabolites [44], counterbalancing oxidative stress and detoxifying enzymes [45,46], non-targeted metabolomics and proteomics have been used to profile a set of inbred lines and hybrid genotypes [47,48], biomarkers identification for drought tolerance [49], identification of hub transcription factors involved in drought stress response in sunflower [50], time-dependent transcriptome analysis to identify drought response mechanisms in sunflower [51], drought response genomic biomarker identification in leaves and roots [52,53], genomic, phenotypic, and physicochemical connections to understand the drought response mechanism in sunflower [54], and phenotypic and transcriptomic responses to cultivated sunflower in multiple abiotic stresses [55,56]. However, the abiotic stress tolerance properties of *MATE* genes in sunflower have not yet been studied, even though they have been discovered in many plants and shown to be SR. For example, there are 66 *MATE* genes in apple [57], 85 in pear [58], 56 in *Arabidopsis*, 46 in rice [36,59], 69 in citrus fruit [60], 138 in tobacco [61], 71 in Populus [62], 49 in maize [63], 67 in tomato [64], 35 in dragon fruit [39], 39 in melon [65], 128, 70, and 72 genes in *Gossypium hirsutum, Gossypium arboreum*, and *Gossypium raimondii*, respectively [66], and 64 in potato [42]. Therefore, in this study, we attempted to identify the *MATE* gene family and explore its role in AST in sunflower.

## 2. Materials and methods

**Fig 1:**
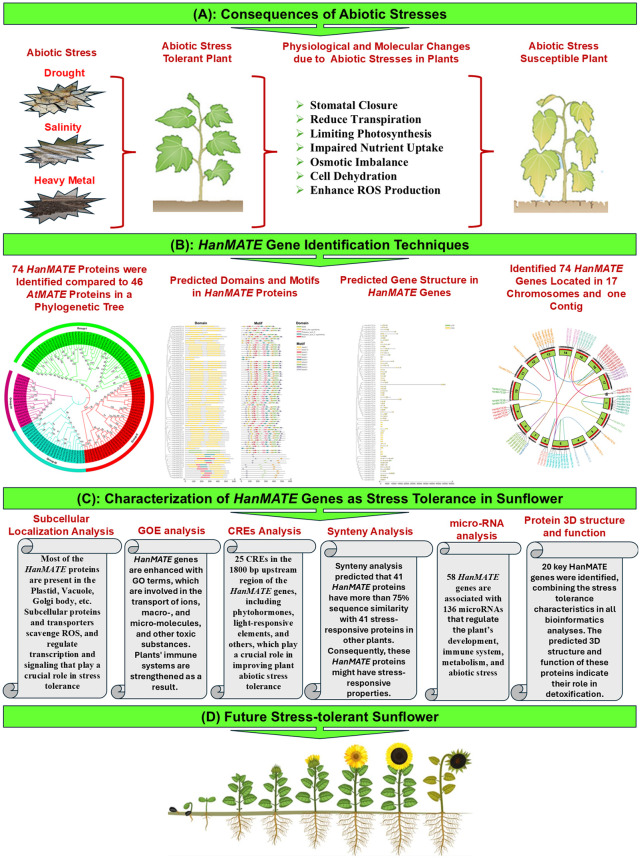
Conceptual and working flowchart for identifying HanMATE genes and their predicted roles in AST in sunflower. The flowchart section (A) outlines the consequences of abiotic stresses on plants. The healthy and stressed plants of this section were obtained from Rani et al. 2021 [67]. Section (B) of the flowchart describes the identification techniques of HanMATE genes. In section **(C)**, the identified HanMATE genes’ AST properties were characterized. In the final section **(D)**, the future stress-tolerant sunflower was depicted; the image was obtained from https://www.dreamstime.com/illustration/sunflower-plant-growth-stages.html.

This section was broadly classified into three categories: sequence and physicochemical data collection, identification of the HanMATE genes, and exploration of their roles in abiotic stress tolerance. A conceptual and working flowchart for identifying HanMATE genes and their predicted roles in AST in sunflower is visualized in Fig 1. Ethical approval is not applicable for this study.

### 2.1. Sequence and physicochemical data collection of *HanMATE* genes

#### 2.1.1. Sequence data collection.

To investigate the *HanMATE* genes, we used the sunflower (*Helianthus annuus* r1.2) Genome’s [68] gene, protein coding sequence (CDS), and proteome sequences, which were collected from the Phytozome Database [69] with Phytozome genome ID: 494, NCBI taxonomy ID: 4232, and website: https://phytozome-next.jgi.doe.gov/info/Hannuus_r1_2. The 46 *AtMATE* protein sequences that were downloaded from the *Arabidopsis* Information Resource (TAIR) database [70] (https://www.arabidopsis.org/) used as query sequences to explore the *HanMATE* gene, protein, and CDS sequences using the BLASTP [71] search. We mainly consider *HanMATE* genes that have an identity score > 30%, a bit score > 50, and an E-value < 10e − 10, as these provide a more trustworthy homology [72,73]. However, the sequence duplication was prevented considering the primary sequence exclusively.

#### 2.1.2. Data collection on basic physicochemical properties.

We leveraged the Phytozome database to obtain the *HanMATE* genes’ genomic length, protein ID, CDS length, and encoded protein length. The ExPASy database was utilized to ascertain the significant protein sequences’ grand average of hydropathicity (GRAVY), isoelectric point (pI), protein length (aa), and molecular weight (MW) [74].

### 2.2. Identification of the *HanMATE* genes

#### 2.2.1. Phylogenetic analysis.

This study used the integrated tools MEGA11 [75] and iTOL [76] to construct the phylogenetic tree. The ClustalW program aligned protein sequences for 74 *HanMATE* and 46 *AtMATE* members. The phylogenetic tree was then built using the neighbor-joining algorithm, with the bootstrap replicate value set at 1000.

#### 2.2.2. Domain, motif, and gene structure analysis.

The conserved domain structure of the 74 *HanMATE* protein sequences was constructed using Pfam [77] and TBtools [78]. Motifs within the *HanMATE* proteins were identified using the MEME Suite [79] database (https://meme-suite.org/meme/tools/meme) and Tbtools, with the default settings of motif width between 6 and 50 (inclusive) and the maximum number of motifs being 10. Gene structure analysis was performed using the Gene Structure Display Server (GSDS) [80] (https://gsds.gao-lab.org/) and Tbtools.

#### 2.2.3. Chromosomal location and gene duplication analysis.

Tbtools illustrated the chromosomal locations of the 74 *HanMATE* genes. The linked gene was determined using MEGA 11 software. TBtools software was also used to create a schematic diagram of positional relationships among the collinear genes.

### 2.3. Role of *HanMATE* genes in AST

#### 2.3.1. Sub-cellular localization analysis.

The subcellular location of certain proteins regulates the biological functions of plant cells. In conjunction, a web-based application, WoLF PSORT [81] (https://wolfpsort.hgc.jp/), was employed to predict the likelihood of *HanMATE* proteins within cells’ membrane-bound compartments/organelles. However, the co-cluster analysis simultaneously clusters the row (*HanMATE* proteins) and column (organelles) entities of a data matrix. Consequently, *HanMATE* proteins and organelles cluster together based on the strength of their association [82]. Finally, the R package “rhcoclust” [83] was utilized to analyze the relationship between *HanMATE* proteins and cell organelles based on the WoLF PSORT data. The rhcoclust is a robust approach to hierarchical co-clustering [84] obtained from the logistic transformation of data, which converts the original data into a range from 0.00 to +1.00. However, this study customized this range into WoLF PSORT-generated data.

#### 2.3.2. The gene ontology enrichment (GOE) analysis.

The GOE analysis of the 74 *HanMATE* genes to the biological processes, molecular functions, and cellular components was analyzed using the online platform agriGO v2.0 [85] (https://systemsbiology.cau.edu.cn/agriGOv2/).

#### 2.3.3. CREs in the promoter regions of *HanMATE* genes.

The 1.8 kbp upstream sequence of the translation initiation codon of the *HanMATE* and abiotic SR (ASR) *MATE* genes in other plants (potato, soybean, rice, cotton, *Arabidopsis*, dragon fruit, wheat, and maize) was curated from the Phytozome database. The CREs in the 1.8 kbp upstream region were then predicted using the web tool PlantCARE [86] (https://bioinformatics.psb.ugent.be/webtools/plantcare/html/). The frequency of presence of CREs in the 1.8 kbp upstream region of *HanMATE and* ASR *MATE* genes was visualized using the hierarchical co-clustering algorithm [82,83].

#### 2.3.4. Syntenic relationship analysis of *HanMATE* genes with other plants’ ASR *MATE* genes.

The syntenic relationship of the ASR *MATE* genes from different plant species with the *HanMATE* gene was analyzed to predict the functional roles of *HanMATE* genes in AST. A Circos diagram between *HanMATE* and ASR *MATE* proteins was drawn with >75% homology, using the web tool Circoletto [87] (https://bat.infspire.org/circoletto/).

#### 2.3.5. Regulatory Network with microRNAs (miRNAs).

In plants and animals, miRNAs are single-stranded noncoding RNA molecules of 19–24 nucleotides (nt). The miRNAs control plant growth, development, and stress response, regulating gene expression at the transcriptional and posttranscriptional levels [88–91]. In this section, we used the Plant miRNA Encyclopedia (PmiREN) [92] (https://www.pmiren.com/download) to analyze the association between miRNA and *HanMATE* genes using mature miRNA sequences and their expression in sunflower. The interaction between the miRNAs and *HanMATE* genes was then visualized using Cytoscape 3.7.1.

#### 2.3.6. 3D structural modeling, functional prediction, molecular docking, and dynamic simulations.

To explore the structural and functional properties of *HanMATE* proteins, three-dimensional (3D) models were generated using the SWISS-MODEL web server [93]. Homology-based modeling offered insights into potential protein conformations and functional roles. Nonetheless, secondary metabolites play a crucial role in plants’ adaptation to abiotic stress through physiological and biochemical mechanisms. Among these metabolites, flavonoids regulate plant growth and development, hormone signaling, and the maintenance of ROS homeostasis [94,95]. We retrieve the molecular 3D structure of the important flavonoids from the online PubChem database [96]. The *HanMATE* proteins and flavonoids were pre-processed using AutoDock tools [97], and then MD analysis between *HanMATE* receptors and compounds was performed, and their binding affinity scores (kcal/mol) were computed using AutoDock Vina [98]. To explore the dynamic characteristics of the best protein-flavonoid complexes, we performed molecular dynamics simulations using the YASARA software [99].

## 3. Results

The findings were then separated into two primary sections: results related to identifying the *HanMATE* genes and their role in AST in sunflower.

### 3.1. Identification of *HanMATE* genes

#### 3.1.1. *HanMATE* gene identification using phylogeny, domain, and motif analysis.

**Fig 2:**
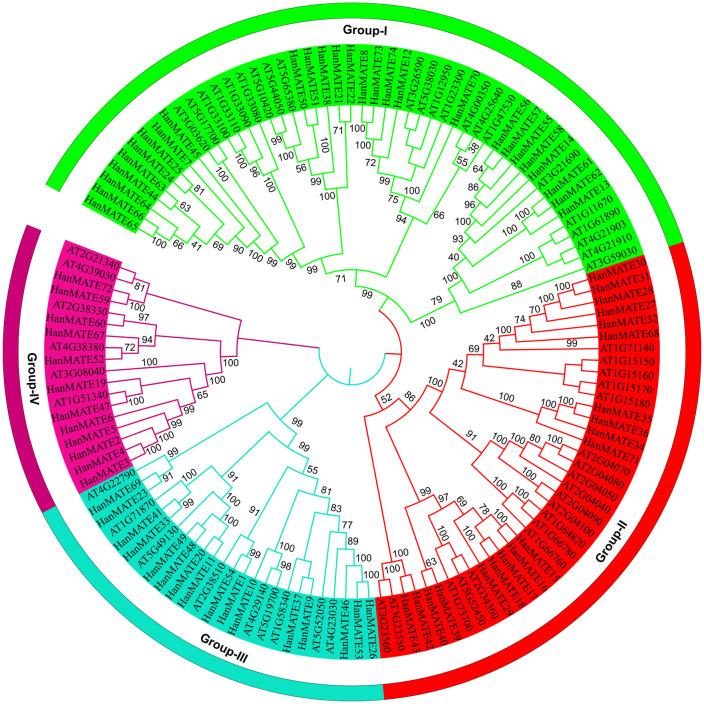
The phylogenetic relationship of the HanMATE and AtMATE proteins in sunflower and Arabidopsis, respectively. The colors in the figure represent different subgroups of HanMATE along with AtMATE proteins. Accordingly, we found four subgroups (I–IV) of 74 HanMATE and 46 AtMATE proteins in the figure.

**Fig 3:**
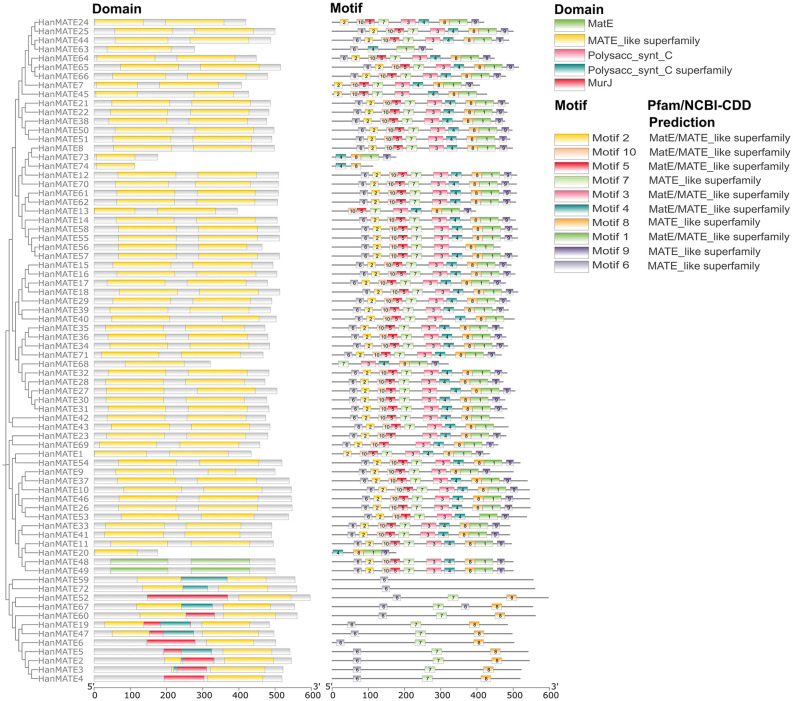
Domain and motif analysis of the HanMATE proteins. The horizontal line at the bottom of each figure represents the protein’s length (0-600 aa).

The best candidate *HanMATE* genes were identified by comparing the phylogenetic relationship between the candidate *HanMATE* and *AtMATE* proteins, considering 1000 bootstrap replicates. Accordingly, 74 *HanMATE* proteins and 46 *AtMATE* proteins were categorized into four clades (I–IV) (Fig 2). All *HanMATE* proteins have the conserved domain *MATE*-like superfamily except *HanMATE* 48 and 49, which have the MatE conserved domain. *HanMATE* 2, 3, 5, 19, 47, 59, 67, and 72 proteins have the conserved domain Polysacc_synt_C along with the *MATE*-like superfamily. In contrast, *HanMATE* 2, 3, 4, 5, 6, 19, 47, 52, and 60 have the conserved domain MurJ (Fig 3). Additionally, evolutionary relationships within *HanMATE* protein groups were investigated using 10 conserved motifs in each *HanMATE* protein. As we observed from Fig 3, these 10 conserved motifs were found in 45 *HanMATE* proteins. *HanMATE* 10, 3, 4, and 8 proteins consist of 9, 8, 4, and 3 motifs, respectively. *HanMATE* 59 and 72 have a single motif. Only 2 motifs were found in *HanMATE*74, while 6 motifs were found in *HanMATE*68. These analyses help to understand the evolutionary relationships of closely related proteins within the group. The protein sequences of the 74 *HanMATE* genes were provided in supplementary file in S1 File.

#### 3.1.2. Basic physicochemical properties of the *HanMATE* genes.

The basic physicochemical properties of all 74 identified *HanMATE* genes were studied, including the number and position of each gene on the chromosome, the starting and ending position of genes, the CDS length, molecular weight, the protein length, the protein pI, and GRAVY. Data from these properties were summarized in Table 1. The coding region of the *HanMATE* genes ranges from 528 bases (*HanMATE20*: HanXRQChr05g0160891) to 1791 bases (*HanMATE53*: HanXRQChr14g0459011). The protein ranged from 176 (*HanMATE20*: HanXRQChr05g0160891) to 597 (*HanMATE52*: HanXRQChr14g0459011) aa. The protein’s molecular weight ranges from 18707.89 Da (*HanMATE20*: HanXRQChr05g0160891) to 63887.8 Da (*HanMATE52*: HanXRQChr14g0459011). All *HanMATE* proteins are polar (positively charged), since the GRAVY value of these proteins is above 0, ranging from 0.328 (*HanMATE63*:HanXRQChr16g0521421) to 0.907 (*HanMATE24*:HanXRQChr06g0168521). The Ip values of *HanMATE* proteins vary from 4.94 (*HanMATE20*: HanXRQChr05g0160891) to 9.65 (*HanMATE52*: HanXRQChr14g0459011), with an average of 7.29.

**Table 1:** Basic physicochemical properties of *HanMATE* genes.

Gene ID	Gene name	Gene location (bp)	Chrom-osome	CDS length (bp)	Protein length (aa)	Molecular weight	pI	Gravy
HanXRQChr01g0000181	*HanMATE1*	1241808..1243110	1	1302	434	46850.11	8.61	0.825
HanXRQChr01g0015611	*HanMATE2*	92177651..92181957	1	1635	545	58152.75	9.01	0.553
HanXRQChr01g0015741	*HanMATE3*	93808400..93814073	1	1566	522	56043.96	8.18	0.553
HanXRQChr01g0015751	*HanMATE4*	93898758..93902360	1	1557	519	55751.49	7.05	0.538
HanXRQChr01g0015771	*HanMATE5*	94504340..94515107	1	1623	541	57181.49	8.47	0.652
HanXRQChr01g0015791	*HanMATE6*	94671175..94675165	1	1506	502	53034.24	7.67	0.619
HanXRQChr01g0017451	*HanMATE7*	104670042..104676151	1	1221	407	44710.82	5.48	0.809
HanXRQChr02g0040191	*HanMATE8*	54302695..54310213	2	1494	498	54300.24	6.65	0.804
HanXRQChr02g0046891	*HanMATE9*	119502119..119505044	2	1500	500	54484.28	5.92	0.578
HanXRQChr03g0061041	*HanMATE10*	4969486..4971121	3	1635	545	58778.01	8.73	0.499
HanXRQChr03g0089511	*HanMATE11*	157570858..157572653	3	1485	495	54267.45	7.89	0.628
HanXRQChr04g0105641	*HanMATE12*	58356960..58367251	4	1530	510	55600.78	8	0.743
HanXRQChr04g0125551	*HanMATE13*	172266920..172269883	4	1188	396	43487.57	8.11	0.865
HanXRQChr05g0129561	*HanMATE14*	2454718..2459369	5	1518	506	54329.88	6.32	0.7
HanXRQChr05g0137101	*HanMATE15*	46296553..46302253	5	1482	494	53300.15	8.24	0.675
HanXRQChr05g0137111	*HanMATE16*	46302303..46352695	5	1515	505	54789.8	8.41	0.608
HanXRQChr05g0144441	*HanMATE17*	123851695..123855877	5	1437	479	52096.14	8.26	0.81
HanXRQChr05g0144471	*HanMATE18*	124050275..124054457	5	1539	513	55819.43	8.87	0.745
HanXRQChr05g0160281	*HanMATE19*	208253888..208261170	5	1452	484	51631.95	8.55	0.756
HanXRQChr05g0160891	*HanMATE20*	209848290..209848818	5	528	176	18707.89	4.94	0.588
HanXRQChr05g0163211	*HanMATE21*	217178803..217186133	5	1461	487	53414.27	5.99	0.812
HanXRQChr05g0163251	*HanMATE22*	217277750..217281414	5	1449	483	52607.09	6.31	0.776
HanXRQChr06g0164891	*HanMATE23*	2697393..2698833	6	1440	480	52370.59	7.04	0.722
HanXRQChr06g0168521	*HanMATE24*	11176183..11181391	6	1257	419	45692.27	5.94	0.907
HanXRQChr06g0168551	*HanMATE25*	11206681..11211918	6	1500	500	55122.04	5.74	0.755
HanXRQChr07g0206831	*HanMATE26*	101747391..101749032	7	1641	547	59394.46	8.17	0.554
HanXRQChr08g0212991	*HanMATE27*	16036757..16046930	8	1515	505	55389.79	6.15	0.82
HanXRQChr08g0213001	*HanMATE28*	16051168..16054735	8	1416	472	51170.75	7.92	0.768
HanXRQChr08g0213011	*HanMATE29*	16057388..16062580	8	1473	491	53411.17	5.22	0.704
HanXRQChr08g0213021	*HanMATE30*	16072531..16077607	8	1431	477	51847.49	6.6	0.739
HanXRQChr08g0213031	*HanMATE31*	16086352..16091482	8	1449	483	52452.17	6.25	0.702
HanXRQChr08g0213051	*HanMATE32*	16108199..16113465	8	1449	483	52317.99	6.89	0.693
HanXRQChr09g0260641	*HanMATE33*	153532958..153536874	9	1473	491	53467.21	8.39	0.601
HanXRQChr09g0262481	*HanMATE34*	160288453..160291998	9	1455	485	53250.34	8.59	0.699
HanXRQChr09g0262561	*HanMATE35*	160462931..160470762	9	1416	472	51382.82	6.32	0.795
HanXRQChr09g0262581	*HanMATE36*	160480350..160496476	9	1440	480	52565.2	8.4	0.769
HanXRQChr10g0277591	*HanMATE37*	889615..891731	10	1617	539	58557.35	7.86	0.577
HanXRQChr10g0277981	*HanMATE38*	2401093..2412243	10	1431	477	51982.53	6.3	0.847
HanXRQChr10g0296121	*HanMATE39*	117467754..117470600	10	1461	487	52863.66	8.71	0.685
HanXRQChr10g0296141	*HanMATE40*	117475519..117478253	10	1509	503	56196.15	7.84	0.484
HanXRQChr11g0326031	*HanMATE41*	24848370..24853966	11	1470	490	53044.56	7.46	0.642
HanXRQChr12g0364121	*HanMATE42*	33029299..33033653	12	1422	474	51838.25	6.74	0.777
HanXRQChr13g0389251	*HanMATE43*	14653818..14659781	13	1458	486	53096.01	6.4	0.802
HanXRQChr13g0396761	*HanMATE44*	71505455..71511765	13	1464	488	53588.12	5.62	0.716
HanXRQChr13g0396771	*HanMATE45*	71533202..71534954	13	1281	427	47010.86	5.19	0.89
HanXRQChr13g0405621	*HanMATE46*	106962079..106963714	13	1635	545	59277.08	8.01	0.51
HanXRQChr13g0405841	*HanMATE47*	108049704..108057039	13	1491	497	53014.46	8.26	0.704
HanXRQChr13g0418091	*HanMATE48*	167598059..167599691	13	1500	500	54630.83	6.09	0.646
HanXRQChr13g0419251	*HanMATE49*	172005422..172007054	13	1500	500	54630.83	6.09	0.646
HanXRQChr14g0444891	*HanMATE50*	121244220..121249931	14	1491	497	54100.09	6.85	0.816
HanXRQChr14g0444901	*HanMATE51*	121324888..121331175	14	1473	491	53559.47	6.74	0.815
HanXRQChr14g0459011	*HanMATE52*	165715671..165724097	14	1791	597	63887.8	9.65	0.387
HanXRQChr14g0462041	*HanMATE53*	172380405..172382092	14	1611	537	58504.86	7.09	0.435
HanXRQChr15g0463211	*HanMATE54*	998026..1003490	15	1557	519	56380.69	8.62	0.586
HanXRQChr15g0471041	*HanMATE55*	25416771..25420572	15	1536	512	55066.83	7.56	0.66
HanXRQChr15g0471051	*HanMATE56*	25456353..25459229	15	1392	464	49726.89	7.55	0.826
HanXRQChr15g0471091	*HanMATE57*	25667395..25670919	15	1536	512	55052.84	6.75	0.689
HanXRQChr15g0471131	*HanMATE58*	25701725..25705054	15	1536	512	55159.82	6.08	0.664
HanXRQChr15g0479111	*HanMATE59*	57232092..57236533	15	1665	555	59978.26	8.53	0.443
HanXRQChr16g0510301	*HanMATE60*	70086668..70091771	16	1683	561	60076.43	5.8	0.438
HanXRQChr16g0515961	*HanMATE61*	107164753..107171082	16	1527	509	55133.81	6.18	0.681
HanXRQChr16g0516181	*HanMATE62*	108361375..108377280	16	1521	507	55107.14	6.39	0.771
HanXRQChr16g0521421	*HanMATE63*	140453330..140465261	16	831	277	30421.33	7.69	0.328
HanXRQChr16g0521431	*HanMATE64*	140469519..140482836	16	1344	448	49369.6	7.58	0.846
HanXRQChr16g0525361	*HanMATE65*	156928901..156933745	16	1545	515	56790.85	7	0.597
HanXRQChr16g0525441	*HanMATE66*	156988232..156991865	16	1437	479	52758.15	5.9	0.702
HanXRQChr16g0526901	*HanMATE67*	162982284..162987786	16	1662	554	58724.53	8.89	0.512
HanXRQChr16g0529511	*HanMATE68*	172398818..172402057	16	963	321	34870.23	5.96	0.765
HanXRQChr17g0533221	*HanMATE69*	340578..341952	17	1374	458	49785.65	8.93	0.817
HanXRQChr17g0539761	*HanMATE70*	21674612..21679927	17	1527	509	55437.84	5.87	0.598
HanXRQChr17g0543471	*HanMATE71*	34358309..34361459	17	1401	467	50376.05	8.87	0.849
HanXRQChr17g0543631	*HanMATE72*	34970015..34979783	17	1680	560	61042.06	8.1	0.369
HanXRQChr00c0397g0574591	*HanMATE73*	2160..3553	**Contig**	528	176	19416.95	9.51	0.578
HanXRQChr00c0397g0574601	*HanMATE74*	18939..20138	**Contig**	528	112	12185.43	9.44	0.873

#### 3.1.3. Gene structure analysis.

**Fig 4:**
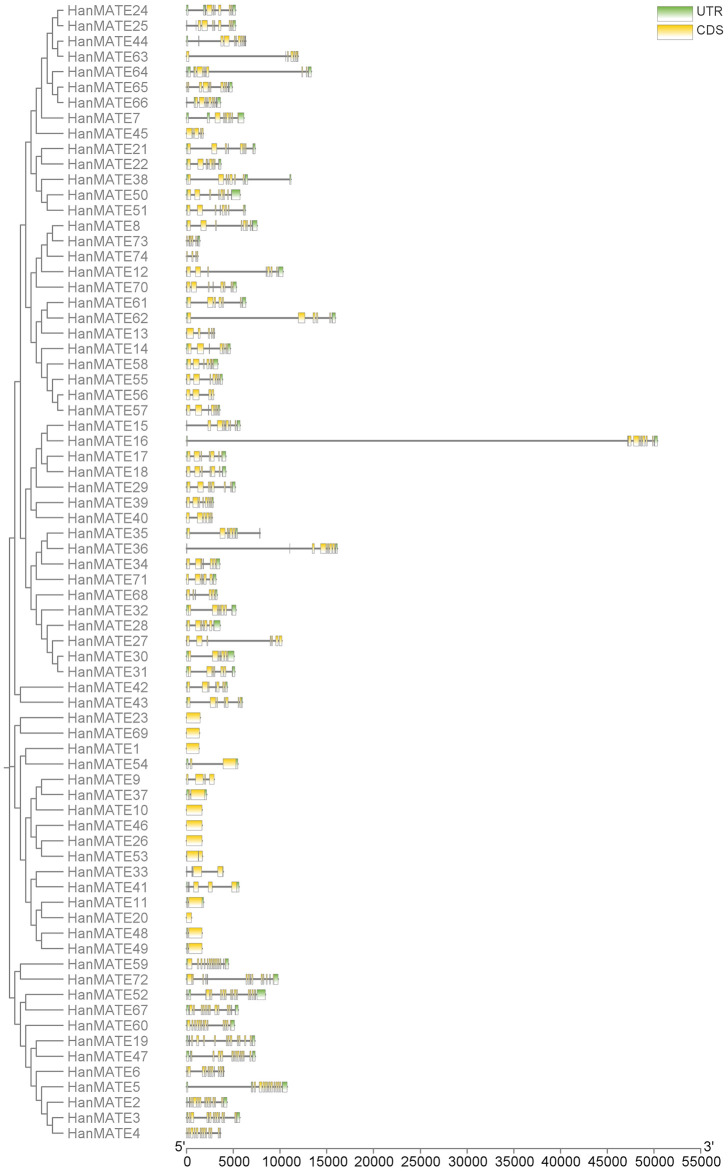
*HanMATE* gene structure analysis. The horizontal line at the bottom of the figure represents the gene’s length (0-55000 bp).

The *HanMATE* gene’s structure was examined by arranging exons and introns, which were identified by comparing the CDS sequences with their corresponding genomic sequences. The *HanMATE* genes were found to contain 1–17 exons and 0–16 introns (Fig 4). Additionally, the evolutionary tree’s gene clusters exhibit almost similar exon and intron structures, based on the number of exons and introns (Fig 2 and Fig 4). For instance, Group I has between 4 and 11 exons and 3–10 introns; Group II has 6–11 exons and 5–10 introns; Group III has 1–5 exons and 0–4 introns; and Group IV has 12–17 exons and 11–16 introns. Nevertheless, 1–17 exons and 0–16 introns are present in the *HanMATE* genes, with the lowest, median, mode, 75th percentile, and maximum exon counts being 1, 9, 9, 10, and 17, in that order (Fig 4). The *HanMATE* gene and CDS sequences are provided in files S2 and S3 in S1 File.

#### 3.1.4. Chromosomal location and gene duplication analysis.

**Fig 5:**
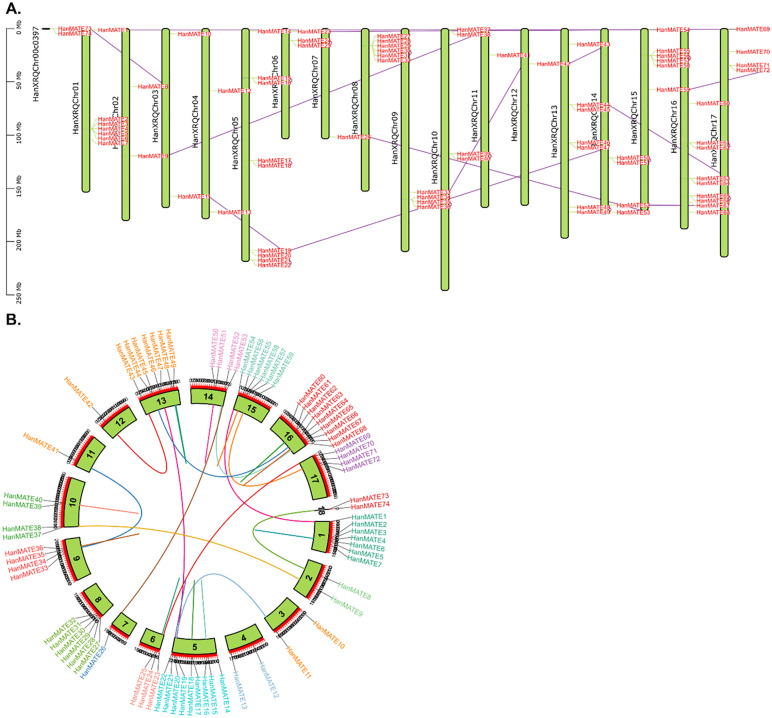
Mapping HanMATE genes over 17 sunflower diploid chromosomes and a contig. In the figure, plot A displays the chromosome number on the left side and the HanMATE name on the right side for each of the chromosomes. Plot B showed HanMATE gene duplication occurrences within the genome. Duplicated gene pairs in sunflower relate to lines, and inside the chromosome numbers are stated.

These chromosomal locations are shown in Fig 5A, where the 74 *HanMATE* genes are distributed across 17 chromosomes and one contig. Chromosomes 5 and 16 contain the maximum number of *HanMATE* genes, with nine each; specifically, *HanMATE* 14–22 is on chromosome 5, and HanMATE 60–68 is on chromosome 16. Chromosomes 1 and 13 each have 7 *HanMATE* genes, with *HanMATE* 1–7 on chromosome 1 and *HanMATE* 43–49 on chromosome 13. Chromosomes 2, 3, and 4, along with one contig, each contain two *HanMATE* genes. Chromosomes 6, 14, 15, and 17 have 3, 4, 6, and 4 *HanMATE* genes, respectively (Fig 5A). Tandem duplicated genes contribute significantly to plant evolution and adaptation to environmental changes. The duplicated genes within or between chromosomes were marked with red lines in Fig 5B. A total of 22 pairs of duplicated *HanMATE* genes were identified, with 11 pairs duplicated between chromosomes and 11 pairs duplicated within the same chromosome (Fig 5B). For example, *HanMATE26* and *HanMATE53* were duplicated on chromosomes 7 and 14, while *HanMATE39* and *HanMATE40* were duplicated within chromosome 10 (Fig 5B).

### 3.2. Role of *HanMATE* genes in AST in sunflower

#### 3.2.1. Subcellular localization analysis.

**Fig 6:**
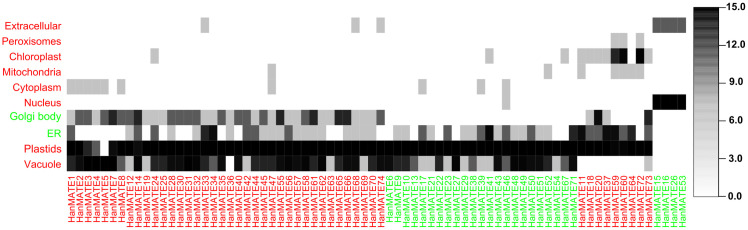
Predicted likelihood of the HanMATE proteins in the subcellular localization.

Proteins’ subcellular location is connected to eukaryotic cells’ biological functions. The placement of proteins within cells helps to explain their functional activities at the cellular level [100,101]. The HanMATE proteins were in the nucleus, vacuole, chloroplast, endoplasmic reticulum (ER), plastid, and Golgi body (Fig 6 and Table 2). On the right side of the figure, a color scale bar ranging from white to black shows the likelihood of HanMATE proteins in relation to cellular organelles; white denotes the absence of HanMATE proteins, and the intensity of black signifies the likelihood. Subsequently, the vacuoles, plastids, ER, Golgi body, nucleus, cytoplasm, mitochondria, chloroplast, peroxisomes, and extracellular organelles contained 62, 69, 48, 42, 5, 10, 7, 11, 3, and 7 of the 74 HanMATE proteins (Table 2).

**Table 2:** Predicted *HanMATE* proteins in the subcellular organelle.

Subcellular Organelle	*#* of *HanMATE* Proteins	Subcellular Organelle	*#* of *HanMATE* Proteins
Vacuole	62	Cytoplasm	10
Plastids	69	Mitochondria	7
ER	48	Chloroplast	11
Golgi Body	42	Peroxisomes	3
Nucleus	5	Extracellular	7

#### 3.2.2. GOE analysis of *HanMATE* genes.

**Fig 7:**
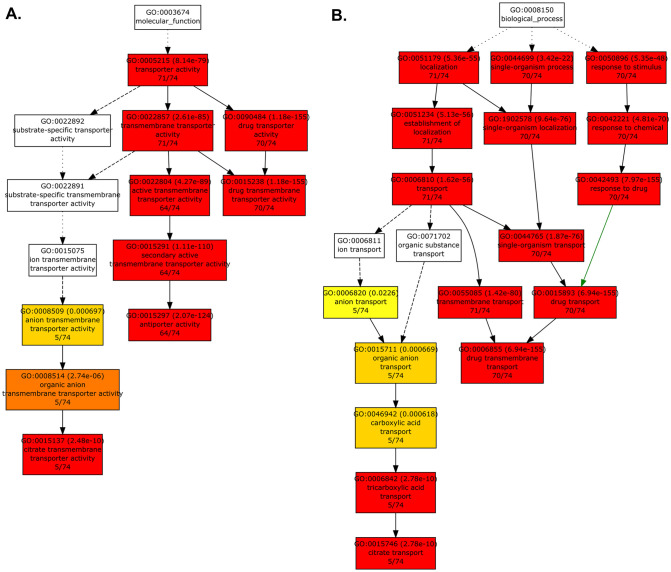
HanMATE gene GO enrichment analysis. Plot A in the figure illustrates the enrichment of HanMATE genes with molecular functions, while plot B shows the enrichment of HanMATE genes with biological processes. Each of the rectangles in the plot shows the GO ID with p-value, the GO term, and the number of HanMATE genes that were enriched for that GO term.

The GOE analysis of the *HanMATE* genes was performed to determine their relationship with various biological processes, molecular functions, and cellular components. Fig 7A and 7B represent the *HanMATE* proteins’ enrichment in the molecular functions and biological processes. According to Fig 7A, 71, 71, 70, 64, 70, 64, and 64 out of 74 *HanMATE* proteins significantly enriched the molecular functions of transporter activity (GO:0005215, p-value = 0.000), transmembrane transporter activity (GO:0022857, p-value = 0.000), drug transporter activity (GO:0090484), active transmembrane transporter activity (GO:0022804, p-value = 0.000), drug transmembrane transporter activity (GO:0015238, p-value = 0.000), secondary active transmembrane transporter activity (GO:0015291, p-value = 0.000), and antiporter activity (GO: 0015297, p-value = 0.000), respectively. Additionally, 5 *HanMATE* proteins were also significantly enriched in the molecular fluctuations of anion transmembrane transporter activity (GO:0008509, p-value = 0.001), organic anion transmembrane transporter activity (GO:0008514, p-value = 0.000), and citrate transmembrane transporter activity (GO:0015137, p-value = 0.000). On the other hand, 71, 71, 70, 71, 70, 70, 71, 70, 70, 71, 70, and 70 out of 74 *HanMATE* genes were significantly enriched in the biological processes such as localization (GO:0051179, p-value = 0.000), single-organism process (GO:0050896, p-value = 0.000), establishment of localization (GO:0051234, p-value = 0.000), single-organism localization (GO:1902578, p-value = 0.000), response to chemical (GO:0042221, p-value = 0.000), transport (GO:0006810, p-value = 0.000), response to drug (GO:0042493 p-value = 0.000), single-organism transport (GO:0044756), transmembrane transport (GO:0055085, p-value = 0.000), drug transport (GO:0015893, p-value = 0.000), and drug transmembrane transport (GO:0006855, p-value = 0.000), respectively. The biological processes like anion transport (GO:0006820, p-value = 0.023), organic anion transport (GO:0015711, p-value = 0.001), carboxylic acid transport (GO:0046942, p-value = 0.001), tricarboxylic acid transport (GO:0006842, p-value = 0.000), and citrate transport (GO:0015746, p-value = 0.000) were significantly enriched with 5 *HanMATE* genes. The detailed results of the GOE analysis were given in file S4 in S1 File.

#### 3.2.3. CREs in the promoter regions of *HanMATE* genes.

**Fig 8:**
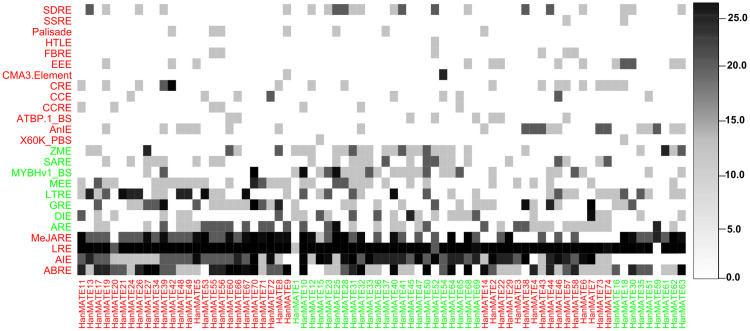
The predicted CREs in the upstream promoter regions of the *HanMATE* genes. In the figure ABRE = Abscisic Acid-Responsive Element, AIE = Anaerobic Induction Element, LRE = Light Responsive Element, MeJARE = MeJA Responsive Element, ARE = Auxin Responsive Element, DIE = Drought Inducibility, GRE = Gibberellin Responsive Element, LTRE = Low Temperature Responsive Element, MEE = Meristem Expression Element, MYBHv1_BS = MYBHv1 binding site, SARE = Salicylic Acid Responsive Element, ZME = Zein Metabolism Element, X60K_PBS = 60K protein binding site, AnIE = Anoxic Inducibility Element, ATBP.1_BS = ATBP-1 Binding Site, CCRE = Cell Cycle Regulation Element, CCE = Circadian Control Element, CRE = Cis Regulatory Element, CMA3 Element = CMA3 Element, EEE = Endosperm Expression Element, FBRE = Flavonoid Biosynthesis Regulation Element, HTLE = High Transcription Level, SSRE = Seed Specific Regulation Element, and SDRE = Stress and Defense Responsive.

**Fig 9:**
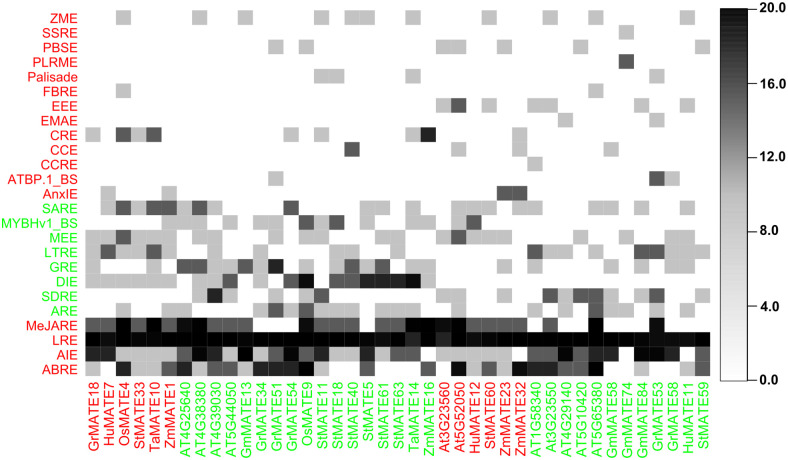
The predicted CREs in the upstream promoter regions of the ASR MATE genes of potato, rice, wheat, maize, soybean, dragon fruit, cotton, and Arabidopsis.

CREs are regions on a gene that can bind to transcription factors. CREs are crucial for gene regulation and control plant growth, development, differentiation, and stress response. In sunflower and other plants, we identified 25 CREs in the 1.8 kbp upstream of the HanMATE and ASR MATE genes, respectively, using the PlantCARE database and co-clustering technique (Figs. 8 and 9). The CREs LRE, MeJARE, ABRE, and AIE were found in the 1.8 kbp upstream region of most HanMATE and ASR MATE genes (Figs. 8 and 9). As a result, ASR MATE and HanMATE genes share 20 of these cis-regulatory elements. CREs for the HanMATE genes are provided in file S5 in S1 File.

#### 3.2.4. Syntenic relationship between *HanMATE* and ASR *MATE* proteins.

**Fig 10:**
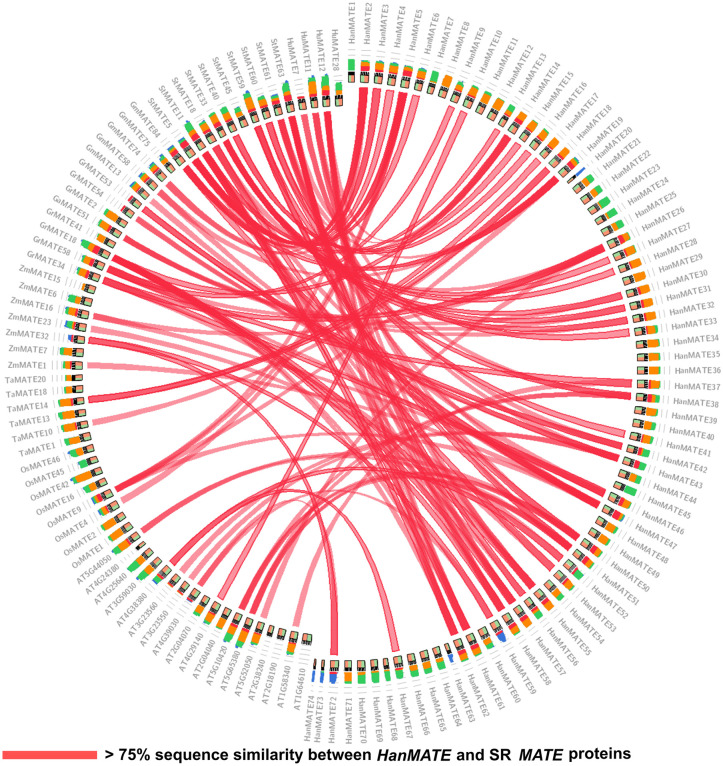
Syntenic relationship analysis of the *HanMATE* proteins with ASR *MATE* proteins in other plants.

Synteny analysis involves large blocks of conserved sequences across genomes, indicated by highly similar patterns between species [102–104]. This study compared 74 *HanMATE* genes in sunflower with 66 ASR *MATE* proteins from different plants, such as *Arabidopsis*, rice, wheat, maize, potato, soybean, and dragon fruit, based on their sequence similarity, as shown in Fig 10. Accordingly, 41 *HanMATE* protein sequences share > 75% sequence similarity with 40 ASR *MATE* proteins (Fig 10), which was summarized in Table 3. The table indicated that seven (*StMATE5/18/33/40/59/60/61*) proteins in potato were sensitive to heavy metal stress, which is associated with 21 *HanMATE2/3/4/5/6/14/15/16/17/27/28/31/32/33/38/41/42/50/51/53/62* proteins. *StMATE11/63* proteins synthesized flavonoid in potato under stress and showed >75% sequence similarity with *HanMATE13/14/26/30/51/61/62* proteins. In soybean, *GmMATE13/58/74/84* proteins are responsive to AL stress that aligned with *HanMATE 2/3/4/5/6/19/26/42/47* proteins. Nevertheless, in rice, *OsMATE4/9* are involved in metal ion detoxification and responsive to salt stress, having >75% sequence similarity with *HanMATE 2/5/19/47* in sunflower. Similarly, six *GaMATE18/34/51/53/54/58* (upregulated in multiple abiotic stresses) and 14 *HanMATE9/14/26/31/32/33/37/42/43/46/57/58/61/62* proteins’ sequences are similar (>75%) in cotton and sunflower, respectively. The sequence of *DTX18/19/45/47/50/51* proteins in *Arabidopsis*, involved in detoxification, transportation, and disease resistance, aligned with that of *HanMATE10/26/37/43/52/55/59/67/72* proteins in sunflower. *DTX48/50* maintain Fe homeostasis in the cell, aligned with *HanMATE50*, which shares >75% sequence similarity. Similarly, *DTX26/28/30* transport Ca^+^ as well as lessen multiple abiotic stresses in *Arabidopsis*, having >75% sequence similarity with *HanMATE50/51*. In contrast, *HuMATE7/11/12* proteins detoxify toxic compounds under heavy metal and salt stresses in dragon fruit, showing >75% sequence similarity with *HanMATE14/38/43/46/55/56/57/58/62*. Conversely, in wheat, *TaMATE10/14* are upregulated under multiple abiotic stresses that share >75% sequence similarity with *HanMATE17/18/61*. However, in maize, *ZmMATE1/16/23/32* proteins are responsive to AL stress and show sequence alignment (>75%) similar to *HanMATE26/46/47/55/56/59/72*.

**Table 3:** *HanMATE* proteins are associated (more than 75% sequence similarity) with various ASR *MATE* proteins in other plants.

SR *MATE* protein	*HanMATE* protein	Related abiotic stress	Reference plant
*StMATE5*	*HanMATE2, HanMATE3, HanMATE4, HanMATE5, HanMATE6*	Responsive to heavy metal (Cu^2+^) stress	Potato [42]
*StMATE18*	*HanMATE13, HanMATE15, HanMATE16, HanMATE53*	Responsive to heavy metal (Cu^2+^and Zn^2+^) stress	Potato [42]
*StMATE33*	*HanMATE27, HanMATE28, HanMATE31, HanMATE32*	Responsive to heavy metal (Cu^2 +^ , and Cd^2+^) stress	Potato [42]
*StMATE40*	*HanMATE15, HanMATE16*	Responsive to heavy metal (Cu^2 +^ , and Zn^2+^) stress	Potato [42]
*StMATE59*	*HanMATE27, HanMATE38, HanMATE50, HanMATE51*	Responsive to heavy metal (Ni^2^) stress	Potato [42]
*StMATE60*	*HanMATE33, HanMATE41*	Responsive to heavy metal (Cu^2+^) stress	Potato [42]
*StMATE61*	*HanMATE14, HanMATE17, HanMATE42, HanMATE62*	Responsive to heavy metal (Cu^2+^) stress	Potato [42]
*StMATE11*	*HanMATE13, HanMATE26, HanMATE30, HanMATE61, HanMATE62*	Flavonoid biosynthesis under stress	Potato [42]
*StMATE63*	*HanMATE14, HanMATE61*	Flavonoid biosynthesis under stress	Potato [42]
*GmMATE13*	*HanMATE42*	Responsive to aluminum stress	Soybean [40]
*GmMATE58*	*HanMATE17*	Responsive to aluminum stress	Soybean [40]
*GmMATE74*	*HanMATE2, HanMATE6*	Responsive to aluminum stress	Soybean [40]
*GmMATE84*	*HanMATE2, HanMATE3, HanMATE4, HanMATE5, HanMATE6, HanMATE19, HanMATE47*	Responsive to aluminum stress	Soybean [40]
*OsMATE4*	*HanMATE5*	Metal ion detoxification and response to salt stress	Rice [41,59]
*OsMATE9*	*HanMATE2, HanMATE5, HanMATE19, HanMATE47*	Metal ion detoxification	Rice [41,59]
*GaMATE51*	*HanMATE31, HanMATE32*	Upregulated in drought, salt, and heavy metal (Cd) stresses	Cotton [38]
*GrMATE18*	*HanMATE14, HanMATE55, HanMATE57, HanMATE58*	Upregulated in drought, salt, and heavy metal (Cd) stresses	Cotton [38]
*GrMATE34*	*HanMATE26, HanMATE46, HanMATE61, HanMATE62*	Upregulated in drought, salt, and heavy metal (Cd) stresses	Cotton [38]
*GrMATE53*	*HanMATE42, HanMATE43*	Upregulated in drought, salt, and heavy metal (Cd) stresses	Cotton [38]
*GrMATE54*	*HanMATE42, HanMATE43*	Upregulated in drought, salt, and heavy metal (Cd) stresses	Cotton [38]
*GrMATE58*	*HanMATE9, HanMATE31, HanMATE32, HanMATE33, HanMATE37*	Upregulated in drought, salt, and heavy metal (Cd) stresses	Cotton [38]
AT3G23550(*DTX18*)	*HanMATE43*	Involved in detoxification, transportation, and disease resistance	*Arabidopsis* [37,59,105–109]
AT3G23560(*DTX19*)	*HanMATE43*	Involved in detoxification, transportation, and disease resistance	*Arabidopsis* [37,59,105–109]
AT4G38380(*DTX45*)	*HanMATE52, HanMATE67*	Involved in detoxification, transportation, and disease resistance	*Arabidopsis* [37,59,105–109]
AT4G39030(*DTX47*)	*HanMATE72, HanMATE59*	Involved in detoxification, transportation, and disease resistance	*Arabidopsis* [37,59,105–109]
AT4G29140(*DTX51*)	*HanMATE10*	Involved in detoxification, transportation, and disease resistance	*Arabidopsis* [37,59,105–109]
AT1G58340(*DTX48*)	*HanMATE38*	Maintain cellular Fe homeostasis by secreting excess Fe	*Arabidopsis* [108–110]
AT5G52050(*DTX50*)	*HanMATE26, HanMATE37*	Maintain cellular Fe homeostasis by secreting excess Fe	*Arabidopsis* [108–110]
AT5G10420(*DTX26*)	*HanMATE50, HanMATE51*	Transport calcium ion (Ca+) and lessen the salt and other abiotic stresses	*Arabidopsis* [66,105,108,111]
AT5G44050(*DTX28*)	*HanMATE50*	Transport calcium ion (Ca+) and lessen the salt and other abiotic stresses	*Arabidopsis* [66,105,108,111]
AT5G65380(*DTX30*)	*HanMATE51*	Transport calcium ion (Ca+) and lessen the salt and other abiotic stresses	*Arabidopsis* [66,105,108,111]
*HuMATE7*	*HanMATE43*	Toxic detoxification under heavy metal and salt stress	Dragon Fruit [39]
*HuMATE11*	*HanMATE55, HanMATE57, HanMATE58*	Toxic detoxification under heavy metal and salt stress	Dragon Fruit [39]
*HuMATE12*	*HanMATE14, HanMATE38, HanMATE46, HanMATE56, HanMATE57, HanMATE58, HanMATE61, HanMATE62*	Toxic detoxification under heavy metal and salt stress	Dragon Fruit [39]
*TaMATE10*	*HanMATE18*	Upregulated under heat, drought, and salt stress	Wheat [112]
*TaMATE14*	*HanMATE17, HanMATE18*	Upregulated under heat, drought, and salt stress	Wheat [112]
*ZmMATE1*	*HanMATE47,*	Responsive to aluminum stress	Maize [63]
*ZmMATE16*	*HanMATE26, HanMATE46,*	Responsive to aluminum stress	Maize [63]
*ZmMATE23*	*HanMATE55, HanMATE56*	Responsive to aluminum stress	Maize [63]
*ZmMATE32*	*HanMATE59, HanMATE72*	Responsive to aluminum stress	Maize [63]

#### 3.2.5. Regulatory network analysis with miRNAs.

**Fig 11:**
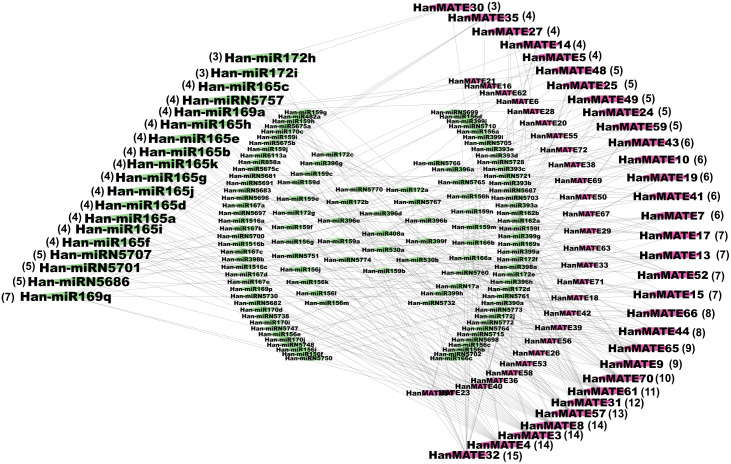
Regulatory network analysis between miRNAs and HanMATE proteins. In the figure, the purple- and green-colored nodes represent the HanMATE proteins and the miRNAs, respectively. The highlighted HanMATE proteins and the miRNAs with interaction scores beside the respective HanMATE proteins and the miRNAs in parentheses are the hub proteins and miRNAs.

In our investigation, we identified 136 miRNAs associated with 58 HanMATE proteins (Fig 11 and File S6 in S1 File). The figure showed that there are 19 major hub miRNAs and 31 important hub HanMTE proteins. The interaction scores of these hub proteins and miRNAs are displayed in parentheses (Fig 11).

#### 3.2.6. Protein 3D structural modeling, functional prediction, and molecular dynamic simulation.

The *HanMATE* proteins were 3D structurally modeled as monomers and exhibited high sequence identity to their respective templates, ranging from 70.92% to 100%, with near-full coverage (≥0.92). The aligned templates were primarily derived from *Helianthus annuus*, *Artemisia annua*, *Mikania micrantha*, *Sesamum indicum*, and other related plant species. Notably, several *HanMATE* proteins, including *HanMATE15/24/25/43/52/61/70*, showed 100% sequence identity and full coverage with *Helianthus annuus* templates, indicating exact or near-exact matches to known sunflower proteins. Functional prediction based on the matched template descriptions indicated that most *HanMATE* proteins are likely involved in protein detoxification or *MATE* efflux transport processes (File S7 in S1 File). Overall, homology-based modeling and functional annotation suggest that *HanMATE* proteins in sunflower are predominantly detoxification-related transporters with highly conserved structure.

**Fig 12:**
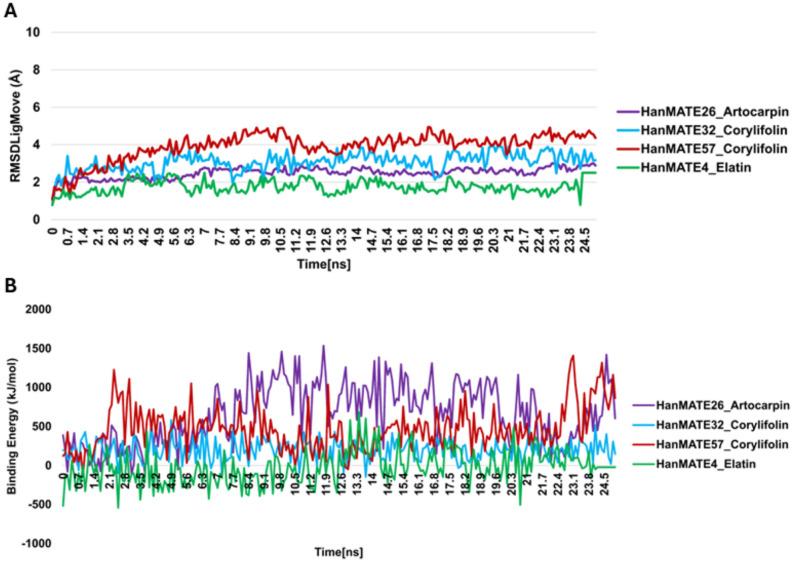
Molecular Dynamics Simulation of *HanMATE* protein and flavonoid complexes. **(A)** Root-mean-square deviation (RMSD) of flavonoid movement. **(B)** Binding free energy (kJ/mol).

MD was performed to assess the binding affinities of nine flavonoids (elatin, corylifolin, artocarpin, laurifolin, anthocyanins, quercetin, and catechin) and citric acid with the randomly selected thirteen *HanMATE* proteins (Table 4). Specific protein-flavonoid interactions highlighted variability among *HanMATE* members. For instance, *HanMATE26* displayed the strongest binding with artocarpin (−9.9 kcal/mol), laurifolin (−9.6 kcal/mol), and Elatin (−9.4 kcal/mol); while *HanMATE32* showed the highest binding affinity with Corylifolin (−9.8 kcal/mol). Other proteins, such as *HanMATE57* and *HanMATE4*, showed preferential binding to Corylifolin (−9.5 kcal/mol) and Elatin (−9.2 kcal/mol), respectively. Overall, these results suggest that artocarpin, corylifolin, laurifolin, and elatin showed consistently high binding affinities for the selected *HanMATE* proteins. These binding affinities were then validated and further explored using molecular dynamics simulations and binding free energy calculations to assess the stability and dynamic behavior of these *HanMATE*-flavonoid complexes. In the molecular dynamic simulations of these complexes, RMSDLigMove (ligand movement RMSD) analysis provided insights into the stability of flavonoid binding within the *HanMATE* protein’s active site (Fig 12A). For *HanMATE4*_Elatin, the RMSD remained low and stable throughout the simulation compared to the other complexes, indicating that the ligand maintained its binding pose with minimal drift inside the pocket. Consistently, the complex exhibited favorable binding affinity with the lowest binding free energy, suggesting strong and stable interactions with the protein (Fig 12B).

**Table 4:** Docking/binding affinity scores (kcal/mol) between the HanMATE proteins (receptors) and flavonoids.

	Elatin	Corylifolin	Artocarpin	Laurifolin	Anthocyanins	Quercetin	Catechin	Ternatin	Citric Acid
**HanMATE26**	−9.4	−8.8	−9.9	−9.6	−8.4	−8.1	−7.8	−7.9	−4.8
**HanMATE32**	−8.1	−9.8	−9.1	−8.8	−9	−8.3	−8.6	−7.3	−4.7
**HanMATE31**	−8.4	−8.9	−8.8	−8.4	−7.8	−8.7	−8.3	−7.1	−5.4
**HanMATE8**	−8.9	−8.7	−8.7	−8.7	−7.7	−8.3	−7.8	−7.6	−5.3
**HanMATE57**	−7.9	−9.5	−7.5	−7.8	−7.8	−8.7	−7.9	−7.2	−5.5
**HanMATE4**	−9.2	−8.5	−8.1	−8.1	−8.4	−7.7	−7.1	−6.9	−5.6
**HanMATE42**	−8.9	−9.1	−8.3	−8.3	−8.5	−7.8	−7.3	−6.5	−4.6
**HanMATE3**	−9.2	−7.9	−8	−8.2	−8.5	−7.7	−7.1	−6.8	−5.5
**HanMATE5**	−9.1	−8.2	−8	−7.8	−8.8	−7.5	−7	−6.9	−5.5
**HanMATE2**	−9.1	−8.1	−8	−8.2	−7.5	−7.5	−7.1	−7	−5.1
**HanMATE6**	−9	−8.3	−8	−8.1	−6.8	−7.8	−7.3	−6.9	−5
**HanMATE19**	−8	−7.9	−8.1	−8.2	−7.1	−7.6	−7.4	−6.7	−4.7
**HanMATE47**	−6.9	−7.9	−7.3	−7.4	−7.7	−6.6	−6.7	−6.4	−4.5

## 4. Discussion

The abiotic stresses of drought, salinity, and heavy metal stress seriously impacted the plant’s growth and development, biodiversity, and productivity [3–5]. These stresses bring morphological, physiological, and molecular changes in plants. Consequently, closing stomata, reducing transpiration, limiting photosynthesis, ionic and osmotic stress, impairing nutrient uptake channels, and excessive production of ROS are the main indications of plants under abiotic stresses [6,7,11,13,15–22]. Nonetheless, the secondary transporter gene, *MATE,* transports substrates like chemical molecules, metabolites, and phytohormones. These transporter proteins promote plant growth and development, detoxify internal and external toxins, and defend prokaryotes and eukaryotes from stresses [27,28,36,102]. On the contrary, the special structure of the root, stem, leaves, and head makes the sunflower moderately AST [43,44]. Therefore, researchers worldwide studied the AST of sunflower in many ways [44–53]. However, researchers have not yet discovered the role of *MATE* genes in sunflower AST, though their functions have been studied in many plants. Therefore, in this study, we tried to find insights into the *MATE* gene family in sunflower for AST.

This study found 74 *HanMATE* genes in sunflower and grouped them into four clades (I-IV). Groups I, II, III, and IV comprise 27, 19, 16, and 12 of the 74 *HanMATE* proteins. The gene, domain, and motif structures of the HanMATE genes are nearly identical, corresponding to the respective phylogenetic groups. For instance, the domain *MATE*-like superfamily is included in Groups I and II. In addition to the domain *MATE*-like superfamily, *HanMATE* proteins in Group III also contain the domain *MATE* (*HanMATE* 48 and 49). In Group IV, the *HanMATE* proteins contain the domains MurJ, *MATE*-like superfamily, and Polyasacc_synt_C superfamily. Likewise, the *HanMATE* genes and proteins organization displayed nearly the same pattern across the various phylogenetic groups. Additionally, the motifs’ distribution over the *HanMATE* proteins shows almost similar patterns in the respective phylogenetic groups. Similarly, in the case of the *HanMATE* gene structure, genes in the same group often have similar structures according to the distribution of exons and introns (Figs 2 and 4). As a result, the genes or proteins in the corresponding evolutionary Groups I, II, III, and IV share nearly identical structures. Conversely, in other plants like melon, soybean, and cotton, similar results were also found [38,40,65].

The domain *MATE* and *MATE*-like superfamily transport secondary metabolites and toxic chemicals across the cell membrane and maintain homeostasis in cells in response to abiotic and biotic stresses [113]. Though the roles of the domains Polyasacc_synt_C and the Polyasacc_synt_C superfamily have not yet been identified, MurJ is crucial for flipping Lipid II across the cytoplasmic membrane in bacteria for biosynthesizing the peptidoglycan [114]. The peptidoglycan precursor flippers MurJ’s homologous genes *MltB* and *VanY* in the moss *Physcomitrella* patens play an important role in chloroplast division [115]. Although chloroplasts are known to carry out photosynthesis, they also enable the assimilation of nitrogen and sulfur as well as the production of amino acids, fatty acids, nucleotides, and hormones in plants [116]. Nevertheless, protein motifs are crucial structural elements, serving as signatures of protein families and aiding in protein function prediction [117]. The 1–10 predicted motifs in *HanMATE* proteins are *MATE* and *MATE*-like superfamily, which are linked with predicted domains responsible for exporting secondary metabolites and toxic chemicals across the cell membrane and maintaining homeostasis in cells in response to abiotic and biotic stresses [113].

Proteins are linear assemblies of amino acids, with their functional properties determined by their physical and chemical characteristics. Usually, most *MATE* proteins consist of 450–550 amino acid residues, while some members with 9–12 transmembrane helices may exceed 700 amino acids. The predicted length of the *HanMATE* protein ranges from 112 to 597 amino acids, with a weight range of 18.70 to 63.88 KD and an Isoelectric Point (Ip) between 4.94 and 7.29. In contrast, rice *MATE* proteins typically range from 370 to 598 amino acids, weigh between 39.41 and 61.65 KD, and have an Ip from 5.01 to 11.98, correlating with responses to drought and salt stresses. Similarly, in maize, *MATE* proteins vary from 125 to 692 amino acids, with a weight range of 13.8 to 73.10 KD, and an Ip from 4.34 to 10.0, responding to aluminum stress. The patterns of *MATE* proteins in dragon fruit are comparable to those in rice, maize, and sunflower, with sizes from 124 to 686 amino acids, weights between 13.19 and 74.37 KD, and Ip values from 5.46 to 8.99, responding to abiotic stresses. Additionally, hydrophilic proteins tend to accumulate in plants under various stress conditions, indicating their protective role. Similarly, dragon fruit *MATE* proteins respond to abiotic stresses with a GRAVY ranging from 0.361 to 0.856, whereas *HanMATE* proteins vary from 0.328 to 0.890. These observations regarding the physicochemical properties of *MATE* proteins sunflower and other plants suggest that these *HanMATE* proteins may play a significant role in AST.

The identified 74 *HanMATE* genes are distributed across seventeen chromosomes and one contig (Fig 5A). These genes are duplicated between or within chromosomes, resulting in the numerous *MATE* genes found in sunflower. The synteny analysis revealed 22 pairs of duplicated *HanMATE* genes, with 11 pairs duplicated between chromosomes and 11 pairs duplicated within chromosomes (Fig 5B). Subsequently, duplicated genes are important in plant evolution and adaptation to environmental changes, including biotic and abiotic stresses [118–121]. Therefore, it could be concluded that the *HanMATE* gene duplication might increase the AST in sunflower.

Abiotic stress significantly impacts plant growth and development by altering subcellular organelles and affecting intracellular compartments. However, subcellular proteins, including transporters, ROS scavengers, and signaling and transcriptional regulators, play a crucial role in stress tolerance [122,123]. These proteins have developed intricate detoxification mechanisms to handle a wide range of potentially toxic compounds, including exogenous xenobiotics and endogenous metabolites, particularly secondary metabolites. Membrane transporters play a vital role in maintaining ionic balance and facilitating the movement of substances across organellar membranes during abiotic stress in plants. They assist in reducing ROS production by regulating ions and metabolites, activating antioxidant enzymes, and improving ROS scavenging [124–126]. After enzymatic modification or synthesis, such compounds are transported and stored in the central vacuoles in plant cells [126]. Nonetheless, nearly 50% of the mitochondrial proteome is dedicated to energy production. Consequently, it generates ATP and ROS through oxidative phosphorylation and the electron transport chain, contributing to plant stress responses, which serve as environmental sensors [127–129]. The inner mitochondrial membrane transporters manage energy and calcium exchange, promoting metabolic stability and enhancing plant resilience to environmental stress [130]. Nevertheless, 74 *HanMATE* proteins were observed in the nucleus, vacuole, chloroplast, endoplasmic reticulum, plastid, and Golgi bodies (Fig 6), which may participate in stress tolerance in sunflower. Almost all *HanMATE* genes were present in the plastids with higher likelihood. Plastids, essential plant cell organelles present in all plant cells and green algae, include chloroplasts, amyloplasts, chromoplasts, and leucoplasts. The components of plastids are responsible for important biological functions. For example, chloroplasts convert CO₂ to carbohydrates, chromoplasts regulate the colors of flowers and fruits, and amyloplasts and leucoplasts store starch and lipids in the cell [131–133]. Plant cells’ vacuoles, which house the majority of the *HanMATE* proteins, are another crucial organelle. It is essential for controlling development and growth, maintaining the equilibrium of the cell’s acidity and pressure, controlling the storage and flow of materials, regulating the mobility and positioning of critical proteins within the cell, and responding to both living and non-living stressors are the primary functions of vacuoles [134,135]. Conversely, the ER is a cell’s organelle that stocks two-thirds of the *HanMATE* proteins. These proteins are involved in signaling and the organism’s or cells’ reaction to stressors and the environment [45,136]. In addition, oxidation, hydroxylation, and deamination of xenobiotics or biological components occur in the ER. Stress-induced H_2_O₂ generation interacts outside of the ER, which triggers antioxidant defense components to balance the cell’s redox state [137–140]. The Golgi apparatus plays important roles in cell wall formation, protein sorting, and glycosylation, which are essential for plant cell development and division [141]. On the other hand, the antioxidants superoxide dismutase, glutathione peroxidases, and catalases are localized in the cytosol, mitochondria, and chloroplasts and scavenge ROS. In *Arabidopsis*, these five ascorbate peroxidases, involved in scavenging ROS generated during photosynthesis [142–144]. Therefore, it could be concluded that *HanMATE* proteins in the subcellular compartment may take part in transporting toxic elements, ROS scavenging, signaling, and transcriptional regulation.

The identified *HanMATE* genes in sunflower were enriched in GO terms relating to molecular function (GO:0003674), biological process (GO:0008150), and cellular component (GO:0005575). Almost all the *HanMATE* genes enriched in the molecular function (GO:0003674) (Fig 7A) participate in the process of directing the movement of substances, including macromolecules, small molecules, and ions, into, out of, within, or between the cells. They also contribute to transferring substances from one side of a membrane to another and the movement of drugs, which can affect an organism’s structure or functioning. They also actively transport a solute across a membrane. The *HanMATE* genes also transfer negatively charged ions, organic anions, and citrate. These genes also play a role in the localization of cells, substances, or cellular entities, such as protein complexes or organelles, through selective degradation [145–147]. Nonetheless, around all 74 *HanMATE* genes enriched to different biological processes significantly (Fig 7B), which are involved in transporting xenobiotics, foreign compounds, into, out of, or within a cell or between cells, is a process that involves agents like transporters or pores. This process can be tethered to or maintained in a specific location and can result in changes in cell or organism activity. Localization of substances or cellular components can also occur through movement, tethering, or selective degradation [146,147].

CREs play a crucial role in gene expression patterning and development, providing the proper spatiotemporal patterning for environmental reactions [148]. On the other hand, phytohormones serve as vital regulators for several physiological and biochemical processes that govern plant growth, development, and productivity in both favorable and stressful conditions [149,150]. The ABRE, AIE, LRE, and MeJARE are important CREs clustered together that are present in the 1.8 kbp upstream region in almost every *HanMATE* and ASR *MATE* genes. ABARE is a crucial phytohormone for plant growth and development, regulating stress responses. It is constantly adjusted in response to physiological and environmental changes. Transcription factors like DREB2A/2B, AREB1, RD22 BP1, and MYC/MYB regulate ABA-responsive gene expression through interactions with CRE such as ABRE and MYCRS/MYBRS [151–153]. In plants, AIEs are DNA sequences that stimulate gene expression in the presence of low oxygen. They serve as binding sites for transcription factors, activating genes to assist plants in adjusting to stress when oxygen levels fall [154–156]. On the other hand, LREs are located within the promoter regions of light-responsive genes, which act as binding sites for proteins that mediate the effects of light on gene expression. In *Arabidopsis*, LREs are involved in the light regulation of the nuclear genes GapA and GapB that encode the A and B subunits of glyceraldehyde 3-phosphate dehydrogenase (G3PDH) [157,158]. G3PDH, a conserved glycolytic enzyme, functions as a moonlighting protein, regulating gene expression, cell signaling, and hormone signaling pathways, thereby influencing plant growth and development [159]. However, the phytohormones, particularly MeJA, salicylic acid, Gibberellin, Auxin, and abscisic acid, can help mitigate the effects of abiotic stresses. MeJA inhibits the uptake of harmful ions and lessens the negative consequences of osmotic stress by controlling either organic or inorganic penetrating ions [160]. According to the current study’s findings, TGACG and CGTCA motifs regulate plant defense against abiotic stressors such as heat, salinity, drought, and cold, and they also have a role in MeJA response [161]. Salicylic acid plays a crucial role in enhancing plant tolerance to abiotic stress by regulating key metabolic processes. Recent research has highlighted the pivotal role of phytohormones in mediating plant responses to environmental changes, encompassing growth, development, and responses to environmental stress [162–164]. Another phytohormone, Gibberellin, regulates various plant functions, including blooming, fruit patterning, root and shoot elongation, and seed germination. Its signaling pathway influences plant growth under stress conditions, and its increased signal transduction promotes tolerance to abiotic stressors [165–168]. Auxin, or indole-3-acetic acid, is another phytohormone that regulates plant growth and development and responds to abiotic stressors. It influences gene expression through auxin response factors, which act as an effector of auxin response and translate chemical signals into gene regulation [169,170]. Consequently, MeJARE, SARE, GRE, ARE, and ABRE, CREs were predicted in the *HanMATE* proteins as well as ASR *MATE* proteins in other plants (Fig 8 and Fig 9) that signify *HanMATE* proteins might work for stress tolerance in sunflower. Among the other CREs in *HanMATE* proteins and stress-responsive *MATE* proteins, circadian control or the circadian clock, responsible for synchronizing environmental signals with biological processes, plays a crucial role in plants’ ability to tolerate and adapt to environmental stress. Stress-related extremes like extreme temperatures, drought, and salinity can lead to crop losses and agricultural limitations. Recent evidence suggests the CCE controls gene expression and stress response hormone signaling [171–173]. However, the evolution of biology is linked to atmospheric dioxygen formation, which reprograms gene expression through regulatory interactions between transcription factors and anaerobic-responsive elements, responding to abiotic stresses like anaerobic, drought, and salt by the conciliation of ABA and ethylene hormones [154]. Sunflower’s AIE could contribute to its ability to endure stress. Plants’ abiotic stress responses and tolerance are greatly influenced by DIE, which controls the expression of several SR genes. Abiotic stressors like heat, cold, salt, and drought are known to be regulated by members of the DIE binding gene family [174–177]. The DIEs are closely connected with the dehydration-responsive element binding regulators [178] and make sunflower AST. FBRE, low-molecular-weight polyphenolic compounds found in plants, have various physiological functions in stress response [179]. The WRKY transcription factors regulate flavonoid biosynthesis during biotic and abiotic stress [180], and since *HanMATE* proteins contain flavonoid biosynthesis regulation CREs in sunflower, they may have stress tolerance properties.

Comparative genomics is a field of biology that predicts homologous genes or proteins based on their functional similarities. In this context, synteny analysis serves as a valuable tool to identify the conservation of homologous genes across species. It helps reveal genome structure and find shared markers in different genomes, offering insights into evolutionary relationships [102,181–185]. In this study, synteny analysis showed that over 75% of the sequences of 41 *HanMATE* proteins and 40 ASR proteins in *Arabidopsis*, rice, maize, potato, cotton, melon, and dragon fruit are similar, suggesting that *HanMATE* proteins may be ASR proteins that play a role in sunflowers’ stress tolerance.

miRNAs are small, non-coding RNAs that regulate gene expression in eukaryotes. They can potentially improve agronomic properties and enhance resistance to abiotic stress in plants. Because changes in miRNA expression in abiotic stress conditions suggest that miRNAs are potential targets for genetic manipulation to engineer AST [186–188]. This study found 19 major hub miRNAs linked to 31 important *HanMATE* genes (Fig 11). Among them, Han-miR169q has been linked with seven *HanMATE* genes. The miR169 regulates abiotic stress responses and plays critical roles in hormone accumulation, antioxidant activity, ion-channeling membrane components, and potential interactions with stress-regulating transcription factors such as AsHsfA and AsWRKYs in *Agrostis stolonifera* L. [189]. ZmmiR169q, a protein in maize, responds to stress-induced ROS signals, reducing their accumulation. Depleting it increases salt tolerance, while overexpressing it decreases. ZmmiR169q repressed the transcript abundance of its target nuclear factor YA8 (ZmNF-YA8), while overexpression of ZmNF-YA8 in maize improved salt tolerance [190]. Although the functions of miR5686, miR5701, miR5701, and miR5757 in abiotic stress responses have not been identified, miRNAs regulate gene expression in eukaryotes and play important roles in AST. However, in Arabidopsis, miRNA165/166 regulates targets, including HD-ZIPIII transcription factors, which in turn influence plant development and stress tolerance through ABA signaling [191,192]. The expression of gma-miR156a and gma-miR172a in chickpea significantly impacts plant morphology and physiology, particularly during reproductive development. These genes regulate multiple traits, potentially enhancing crop yield under changing climatic conditions and responding to heavy metal stress in chickpeas [193,194]. Consequently, 41 of the *HanMATE* proteins matched (>75%) with the 40 ASR *HanMATE* proteins, which are considered for stress tolerance in sunflower (Fig 11 and Table 2).

3D structural and functional prediction suggests that almost all *HanMATE* proteins are detoxification-related transporters in sunflower. However, molecular docking was performed to assess the binding affinities of nine flavonoids (elatin, corylifolin, artocarpin, laurifolin, anthocyanins, quercetin, and catechin) and citric acid with 13 randomly selected *HanMATE* proteins. The protein-flavonoid interactions have strong binding affinity that was validated by molecular dynamic simulation analysis, RMSD, and binding free energy analysis.

## 5. Conclusions

Numerous studies in the literature have examined sunflower abiotic stress tolerance using various methods, including metabolite regulation, oxidative stress defense, enzyme detoxification, and drought response mechanisms. These studies also identified key transcription factors, genomic markers, and phenotypic responses to abiotic stresses. Nevertheless, *MATE* proteins detoxify endogenous and external poisons, strengthening the plant’s immune system and fostering growth and development. These proteins also play an important role in adjusting the ROS inside cells and maintaining cellular homeostasis. However, although the functions of *MATE* proteins have been identified in some other plants, this has not been done yet in sunflower AST. Therefore, this study identified 74 *HanMATE* proteins/genes in sunflower compared with 46 *AtMATE* proteins in *Arabidopsis*. These genes were grouped into four clades in a phylogenetic tree. Comparing the results of domain, motif, gene structure, gene duplication, and physicochemical analysis with other plants, we confirmed that the identified 74 *HanMATE* genes have *MATE*-like properties. The ASR and detoxifying features of *HanMATE* genes were analyzed using sub-cellular localization, microRNA, gene ontology, CREs, syntenic relationship, and protein 3D structure and function, molecular docking, and dynamic simulation analysis. Most of the *HanMATE* proteins are present in the plastid, nucleus, and vacuole with a higher likelihood. The GOE analysis predicted that out of 74 *HanMATE* genes, 71 enriched the molecular functions: transporter activity and drug transporter activity, etc. On the other hand, 70 *HanMATE* genes enriched the biological processes: response to stimulus, response to drugs, and response to chemicals etc. This study also found that ABRE, AIE LRE, and MeJARE are the most important CREs in the 1.8 kbp upstream region of the *HanMATE* genes that play a crucial role in the plant’s immune boosting as well as growth and development. Furthermore, the syntenic relationship analysis revealed that 41 *HanMATE* proteins share more than 75% sequence similarity with 40 ASR *MATE* proteins in other plants, indicating that the *HanMATE* proteins may be ASR and contribute to sunflower’s stress tolerance. On the other hand, miRNAs regulate gene expression, may enhance plant agronomic traits and resistance to abiotic stress, indicating the potential for genetic manipulation to improve AST. This study identified 136 miRNAs associated with 58 *HanMATE* proteins, including 19 major hub miRNAs and 31 key hub *HanMATE* proteins. Additionally, these findings were also validated using protein 3D structure and function prediction based on their homologs in common ancestors, which found that the key *HanMATE* genes may participate in detoxification. Additionally, molecular docking and dynamic simulation validated that *HanMATE* genes have strong binding affinity with flavonoids and citric acid. Besides these *in silico* validations of *HanMATE* genes’ ASR properties, an experimental study could be conducted in the future for wet lab agreement. Consequently, the discovered *HanMATE* genes may be crucial for sunflowers’ ability to withstand abiotic stress and may also be applied to the development of robust, more resilient crops.

## Supporting information

S1 File S1. Protein sequences of *HanMATE* genes. S2. *HanMATE* gene sequences. S3. CDS sequences of *HanMATE* genes. S4. *HanMATE* genes’ GOE analysis results. S5. *HanMATE* genes’ CRE analysis results. S6. *HanMATE* gene and miRNA analysis results. S7. *HanMATE* proteins’ 3D structure and functional prediction results.
(ZIP)
