# Supplementary material for: Genome-wide bioinformatics analysis of the MATE gene family for abiotic stress tolerance in sunflower (Helianthus annuus L.)
Source: PLoS One. 2026 Apr 13;21(4):e0346769. doi: 10.1371/journal.pone.0346769 (PMC13075712; doi:10.1371/journal.pone.0346769)
Supplement: S1 File — S2. HanMATE gene sequences. S3. CDS sequences of HanMATE genes. S4. HanMATE genes’ GOE analysis results. S5. HanMATE genes’ CRE analysis results. S6. HanMATE gene and miRNA analysis results. S7. HanMATE proteins’ 3D structure and functional prediction results. (ZIP) [file pone.0346769.s001.zip › Supporting Information/S7.docx]

| **Gene IDs** | **Matched species** | **Template** | **Seq Identity** | **Oligo-state** | **Found by** | **Method** | **Seq Similarity** | **Range** | **Coverage** | **Description** | **3D protein** |
| --- | --- | --- | --- | --- | --- | --- | --- | --- | --- | --- | --- |
| HanMATE3 | Artemisia annua (Sweet wormwood) | A0A2U1KU77.1.A | 80.04 | monomer | AFDB search | AlphaFold v2 | 0.53 | 1 - 521 | 1.00 | Protein DETOXIFICATION | 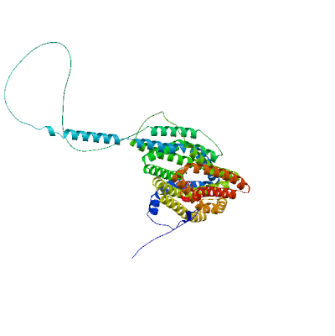 |
| HanMATE4 | Artemisia annua (Sweet wormwood) | A0A2U1KU77.1.A | 79.96 | monomer | AFDB search | AlphaFold v2 | 0.53 | 1 - 518 | 0.99 | Protein DETOXIFICATION | 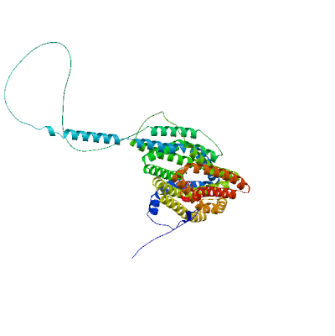 |
| HanMATE5 | Artemisia annua (Sweet wormwood) | A0A2U1KU77.1.A | 86.83 | monomer | AFDB search | AlphaFold v2 | 0.56 | 1 - 540 | 1.00 | Protein DETOXIFICATION | 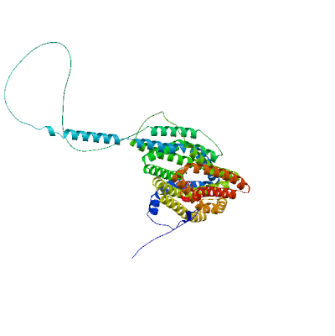 |
| HanMATE7 | Sesamum indicum (Oriental sesame) | A0A6I9SK50.1.A | 71.82 | monomer | AFDB search | AlphaFold v2 | 0.52 | 6 - 406 | 0.99 | Protein DETOXIFICATION | 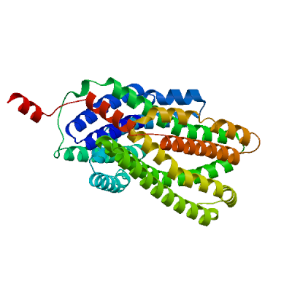 |
| HanMATE8 | Artemisia annua (Sweet wormwood) | A0A2U1P493.1.A | 79.64 | monomer | AFDB search | AlphaFold v2 | 0.55 | 2 - 497 | 1.00 | Protein DETOXIFICATION | 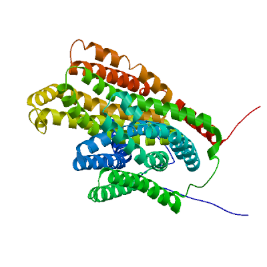 |
| HanMATE9 | Artemisia annua (Sweet wormwood) | A0A2U1P4Y4.1.A | 82.16 | monomer | AFDB search | AlphaFold v2 | 0.55 | 1 - 499 | 1.00 | Protein DETOXIFICATION | 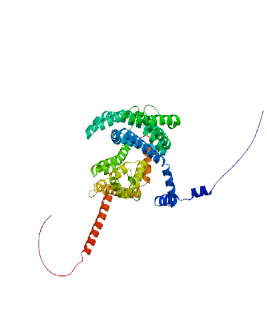 |
| HanMATE10 | Sesamum indicum (Oriental sesame) | A0A6I9UHC5.1.A | 71.35 | monomer | AFDB search | AlphaFold v2 | 0.52 | 1 - 544 | 0.97 | Protein DETOXIFICATION | 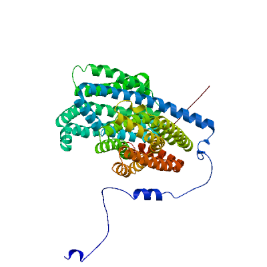 |
| HanMATE13 | Solanum lycopersicum (Tomato) | A0A3Q7EXL3.1.A | 72.96 | monomer | AFDB search | AlphaFold v2 | 0.53 | 1 - 393 | 0.99 | Protein DETOXIFICATION | 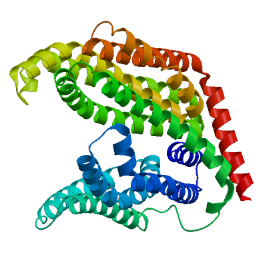 |
| HanMATE14 | Unknown | A0A2J6JVU6.1.A | 83.13 | monomer | AFDB search | AlphaFold v2 | 0.56 | 1 - 505 | 1.00 | Unknown | 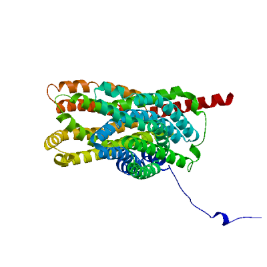 |
| HanMATE15 | Helianthus annuus (Common sunflower) | A0A251UQ57.1.A | 100.00 | monomer | AFDB search | AlphaFold v2 | 0.60 | 1 - 493 | 1.00 | Protein DETOXIFICATION | 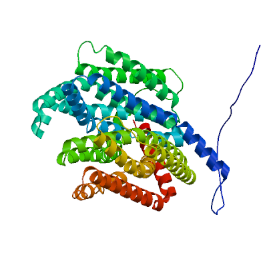 |
| HanMATE17 | Artemisia annua (Sweet wormwood) | A0A2U1L6B3.1.A | 84.73 | monomer | AFDB search | AlphaFold v2 | 0.56 | 1 - 478 | 1.00 | Protein DETOXIFICATION | 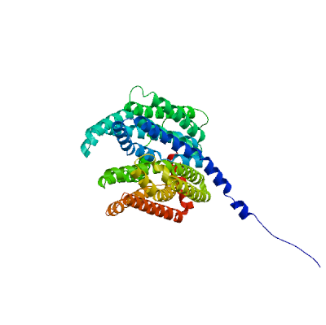 |
| HanMATE19 | Actinidia chinensis var chinensis (Chinese soft-hair kiwi) | A0A2R6R573.1.A | 72.71 | monomer | AFDB search | AlphaFold v2 | 0.51 | 1 - 480 | 0.99 | Protein DETOXIFICATION | 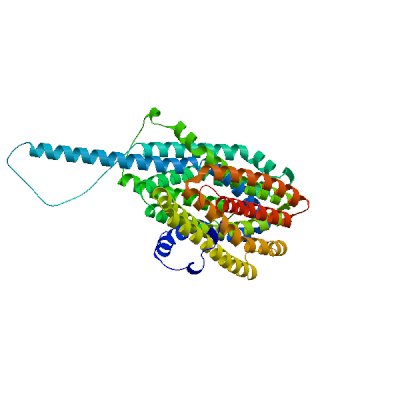 |
| HanMATE24 | Helianthus annuus (Common sunflower) | A0A251UFN4.1.A | 100.00 | monomer | AFDB search | AlphaFold v2 | 0.61 | 1 - 418 | 1.00 | Putative MATE efflux family protein | 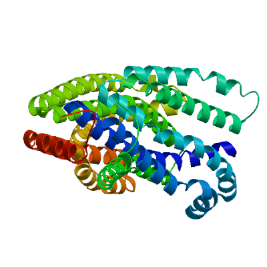 |
| HanMATE25 | Helianthus annuus (Common sunflower) | A0A251UGH9.1.A | 100.00 | monomer | AFDB search | AlphaFold v2 | 0.61 | 1 - 499 | 1.00 | Protein DETOXIFICATION | 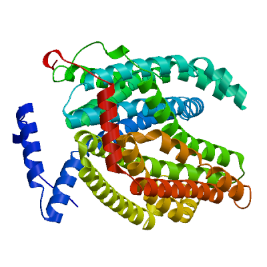 |
| HanMATE27 | Unknown | A0A2J6JNE4.1.A | 74.41 | monomer | AFDB search | AlphaFold v2 | 0.53 | 1 - 495 | 0.92 | Unknown | 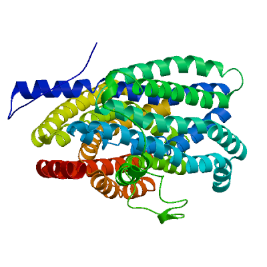 |
| HanMATE30 | Unknown | A0A2J6JNE4.1.A | 80.42 | monomer | AFDB search | AlphaFold v2 | 0.54 | 1 - 475 | 1.00 | Unknown | 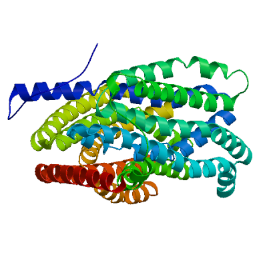 |
| HanMATE31 | Mikania micrantha | A0A5N6LCY9.1.A | 87.97 | monomer | AFDB search | AlphaFold v2 | 0.57 | 1 - 482 | 1.00 | Protein DETOXIFICATION | 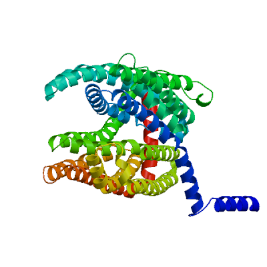 |
| HanMATE32 | Mikania micrantha | A0A5N6LCY9.1.A | 81.84 | monomer | AFDB search | AlphaFold v2 | 0.55 | 1 - 479 | 0.99 | Protein DETOXIFICATION | 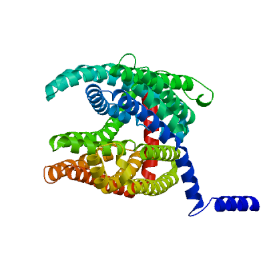 |
| HanMATE35 | Helianthus annuus (Common sunflower) | A0A251TXW7.1.A | 79.62 | monomer | AFDB search | AlphaFold v2 | 0.54 | 1 - 471 | 1.00 | Protein DETOXIFICATION | 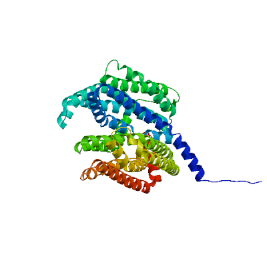 |
| HanMATE41 | Mikania micrantha | A0A5N6LW17.1.A | 89.09 | monomer | AFDB search | AlphaFold v2 | 0.58 | 1 - 487 | 0.99 | Protein DETOXIFICATION | 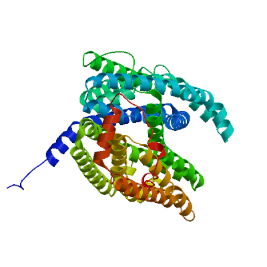 |
| HanMATE43 | Helianthus annuus (Common sunflower) | A0A251SN85.1.A | 100.00 | monomer | AFDB search | AlphaFold v2 | 0.61 | 1 - 485 | 1.00 | Protein DETOXIFICATION | 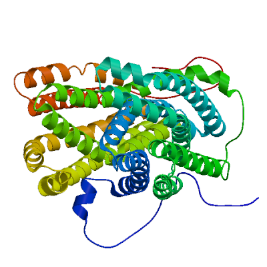 |
| HanMATE44 | Artemisia annua (Sweet wormwood) | A0A2U1KH39.1.A | 84.79 | monomer | AFDB search | AlphaFold v2 | 0.57 | 8 - 487 | 0.99 | Protein DETOXIFICATION | 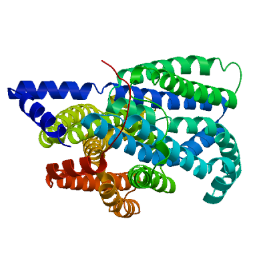 |
| HanMATE48 | Unknown | A0A6S7NPY7.1.A | 79.76 | monomer | AFDB search | AlphaFold v2 | 0.55 | 1 - 499 | 1.00 | Unknown | 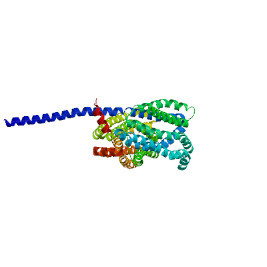 |
| HanMATE49 | Unknown | A0A6S7NPY7.1.A | 79.76 | monomer | AFDB search | AlphaFold v2 | 0.55 | 1 - 499 | 1.00 | Unknown | 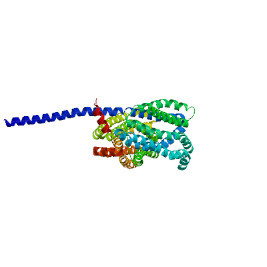 |
| HanMATE52 | Helianthus annuus (Common sunflower) | A0A251SLF2.1.A | 100.00 | monomer | AFDB search | AlphaFold v2 | 0.60 | 1 - 596 | 1.00 | Protein DETOXIFICATION | 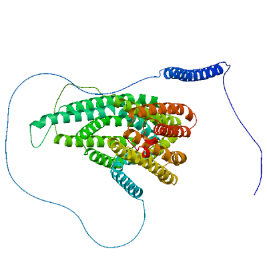 |
| HanMATE57 | Nyssa sinensis | A0A5J4ZDV8.1.A | 73.53 | monomer | AFDB search | AlphaFold v2 | 0.53 | 1 - 511 | 1.00 | Protein DETOXIFICATION | 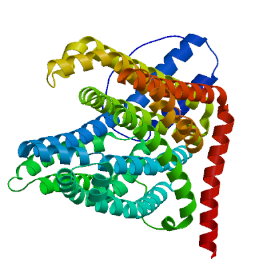 |
| HanMATE59 | Unknown | A0A2J6LVU5.1.A | 84.73 | monomer | AFDB search | AlphaFold v2 | 0.56 | 1 - 553 | 0.99 | Unknown | 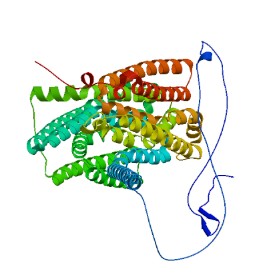 |
| HanMATE61 | Helianthus annuus (Common sunflower) | A0A251RZL8.1.A | 100.00 | monomer | AFDB search | AlphaFold v2 | 0.61 | 1 - 508 | 1.00 | Protein DETOXIFICATION | 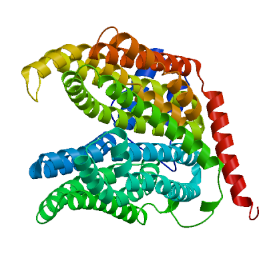 |
| HanMATE65 | Sesamum indicum (Oriental sesame) | A0A6I9SK50.1.A | 70.92 | monomer | AFDB search | AlphaFold v2 | 0.52 | 37 - 514 | 0.93 | Protein DETOXIFICATION | 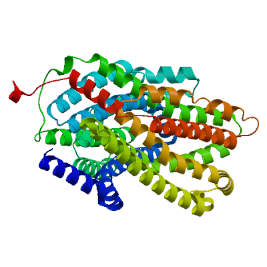 |
| HanMATE66 | Artemisia annua (Sweet wormwood) | A0A2U1KH39.1.A | 74.84 | monomer | AFDB search | AlphaFold v2 | 0.54 | 1 - 477 | 1.00 | Protein DETOXIFICATION | 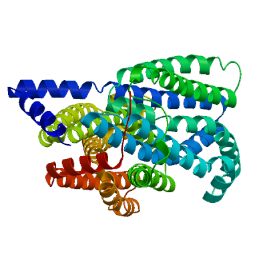 |
| HanMATE70 | Helianthus annuus (Common sunflower) | A0A251RNN1.1.A | 100.00 | monomer | AFDB search | AlphaFold v2 | 0.61 | 1 - 508 | 1.00 | Protein DETOXIFICATION | 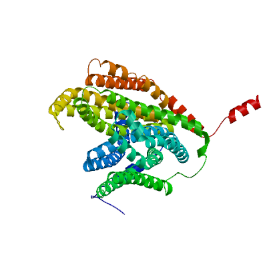 |
